# Phycomediation of cadmium contaminated aqueous solutions using *Chlamydomonas* sp.: process optimization and adsorption characterization

**DOI:** 10.3389/fbioe.2025.1558757

**Published:** 2025-03-26

**Authors:** Reem Mohammed Alharbi, Neveen Abdel-Raouf, Mostafa Shaaban Mohamed, Wael A. Fathy, Ibraheem Borie M. Ibraheem, Walaa Gamal Hozayen

**Affiliations:** ^1^ Biology Department, Science College, University of Hafr Al-Batin, Hafr Al-Batin, Saudi Arabia; ^2^ Department of Biology, College of Science and Humanities in Al-Kharj, Prince Sattam Bin Abdulaziz University, Al-Kharj, Saudi Arabia; ^3^ Botany and Microbiology Department, Faculty of Science, Beni-Suef University, Beni-Suef, Egypt; ^4^ Biochemistry Department, Faculty of Science, Beni-Suef University, Beni-Suef, Egypt

**Keywords:** phycoremediation, biosorption, cadmium (II), industrial wastewater, chlamydomonas

## Abstract

The contamination resulting from heavy metals present in industrial effluents represents a critical global challenge, posing profound risks to aquatic ecosystems and human health. Industrial activities worldwide release wastewater laden with toxic metals, prominently cadmium (Cd), into rivers, lakes, and oceans, frequently surpassing permissible limits. Current treatment technologies are costly and may produce secondary pollutants, thereby necessitating the urgent development of sustainable and cost-effective alternatives. This study investigates the efficacy of the microalga *Chlamydomonas* sp. as a natural biosorbent for the phycoremediation of cadmium from industrial effluent. Critical parameters affecting cadmium biosorption, including pH, contact time, Cd concentration, and biomass dosage, were optimized. Under optimal conditions of 25°C, pH 4, Cd concentration of 50 mg/L, a contact time of 60 min, and a biomass dosage of 0.8 g/L, *Chlamydomonas* demonstrated a cadmium adsorption capacity of 44.75 mg/g, achieving a removal efficiency of 95.6%. Analytical techniques such as SEM, XRD, FTIR, DLS, and zeta potential analysis confirmed cadmium binding to the algal biomass. Kinetic modeling suggested a pseudo-second-order process, while isotherm analysis adhered to the Langmuir model, indicating considerable adsorption capacity and efficiency under optimal conditions. These results support using *Chlamydomonas* as an effective biosorbent for integration into global industrial wastewater treatment systems. This biological approach offers a sustainable and cost-efficient method for heavy metal removal, reducing secondary pollution and aligning with international efforts to mitigate water contamination. Implementing bioremediation strategies could greatly decrease the release of toxic metals into aquatic ecosystems, providing a scalable and environmentally friendly solution for industrial applications globally.

## 1 Introduction

The persistent accumulation of industrial micropollutants in the ecosystem, including heavy metals, dyes, and organic waste, represents a significant threat to human wellbeing and the overall health of global flora and fauna (Narayanan et al., 2022). The utilization of both living and non-living microalgae biomass has recently been acknowledged and extensively applied as prospective agents in micropollutant bioremediation to provide environmentally sustainable solutions (Ratnasari et al., 2022; Fathy et al., 2024). The bioaccumulation of heavy metals predominantly exerts negative impacts on aquatic ecosystems, consequently imposing considerable risks to human health and environmental integrity, as metal ions proceed through the trophic levels of the food chain, they become concentrated in the biological tissues, resulting in the manifestation of diverse diseases (Ashar et al., 2022). Trace amounts of certain heavy metals contribute positively to the metabolic processes of numerous living organisms; nevertheless, elevated concentrations induce detrimental and potentially lethal effects (Sibi, 2014).

Water serves as the fundamental resource essential for sustaining life on Earth and plays a crucial role in a multitude of biological processes. Despite water covering the majority of the Earth’s surface, the accessibility of potable water has consistently decreased in recent years (Kumar Reddy and Lee, 2012). Projections indicate that within the next two decades, approximately two-thirds of the world’s population will experience a freshwater scarcity (Mishra, 2023). To mitigate the global freshwater crisis, it is essential to adopt technologies for wastewater reclamation across the sanitation, agriculture, residential, and industrial sectors (Machineni, 2019).

The heavy metal cadmium (Cd^2+^) constitutes a considerable environmental pollutant, often released in industrial effluents at concentrations ranging from 50 to 200 mg/L, principally originating from electroplating facilities, metal refineries, battery manufacturing plants, and electronic waste processing (Ranade and Bhandari, 2014; Ashfaq et al., 2017). These concentrations significantly surpass the World Health Organization’s prescribed guideline value of 0.003 mg/L for drinking water, established to safeguard consumers from the severe toxicological effects of cadmium (Khwaja and Aslam, 2018). Likewise, the US Environmental Protection Agency has delineated a Maximum Contaminant Level of 0.005 mg/L for cadmium in potable water sources. The marked disparity between industrial discharge concentrations and regulatory limits highlights the urgent necessity for effective remediation technologies. Cadmium represents a chemical element necessitating precise strategies to alleviate its adverse effects on a range of biochemical processes in humans, animals, and other diverse living organisms (Shanker, 2008). In individuals, impact of Cd^2+^ extends beyond renal and osseous systems, influencing every organ and tissue wherein it amasses, this situation necessitates the establishment of comprehensive awareness initiatives to mitigate exposure (Sharma et al., 2015). Several processes might minimize the heavy metal danger in its operations, creating the possibility of reduced metal toxicity involving Cd^2+^ (Abdulkareem and Anwer, 2020). Cadmium also has a particularly hazardous effect on the genetic structure of a human cell, causing apoptosis, oxidative stress, DNA methylation, and DNA damage (Briffa et al., 2020).

Biosorption effectively adsorbs metals by exploiting chemically reactive sites or functional groups present in inert or non-viable cell biomass; indeed, biomass can proficiently sequester or accumulate excessive metals in highly dilute aqueous solutions (Göksungur et al., 2005). Many scientists are intrigued by the possibility of utilizing microorganisms to treat heavy metal contamination. In terms of affordability, nontoxicity, and environmental ecofriendly, they exceed existing procedures (e.g., filtration, coagulation, precipitation, ion exchange, solvent extraction, and adsorption) (Maznah et al., 2012; Zheng et al., 2022). Microalgae, due to their availability and abundance in the surroundings become one of the tools used in heavy metal removal from wastewater (Sibi, 2019). Recently focused studies use microalgae’s ability to reuse heavy metal-contaminated wastewater, one of its benefits is the simplicity of use either in dry dead or living form (Kumar et al., 2015). Another advantage lies in its cost-effectiveness, as the used microalgae can be recycled and applied for biosorption multiple times while ensuring environmental safety (Fekry et al., 2024). Furthermore, microalgae demonstrate considerable efficiency in heavy metal removal, rendering them a preferable alternative to other environmentally detrimental chemical methods (Inthorn et al., 2002).

This study seeks to investigate the phycoremediation potential of *Chlamydomonas* in the treatment of cadmium-contaminated water, with a concentrated focus on optimizing procedures and characterizing adsorption processes. *Chlamydomonas* sp. was chosen above other prevalent microalgae, such as *Spirulina* or *Chlorella*, owing to its enhanced cadmium biosorption capacity and several beneficial biological characteristics. Previous research indicated that *Chlamydomonas* species predominantly employ a chemisorption method for cadmium sequestration, facilitating robust and stable metal binding (Yongbin et al., 2010). The improved performance is due to its elevated surface-area-to-volume ratio and the prevalence of functional groups, especially carboxyl (-COOH) and hydroxyl (-OH) groups, which act as principal cadmium-binding sites. Furthermore, *Chlamydomonas* sp. has remarkably rapid metal uptake kinetics, with research suggesting nearly immediate cytoplasmic equilibration of heavy metals. In addition to its biosorption efficacy, *Chlamydomonas* sp. presents practical benefits, such as its extensive availability, resilience to extreme environmental conditions (including fluctuating pH, temperature, and salinity) (Arora et al., 2025), and rapid growth rate, rendering it a formidable and scalable option for cadmium remediation in aquatic environments (Rojas-Villalta et al., 2024). This research endeavors to discern the optimal conditions required for maximizing cadmium removal efficiency and to elucidate the underlying mechanisms of cadmium adsorption. By integrating batch experiments with sophisticated spectroscopic techniques, this study delineates the kinetics, isotherms, and thermodynamics associated with cadmium binding to *Chlamydomonas*, thereby providing a thorough understanding of its remediation potential. The projected outcomes of this research are anticipated to contribute to the formulation of scalable and sustainable approaches for mitigating cadmium contamination in wastewater, aligning with global initiatives aimed at safeguarding water resources and enhancing environmental health.

## 2 Materials and methods

### 2.1 Biosorbent preparation

**FIGURE 1:**
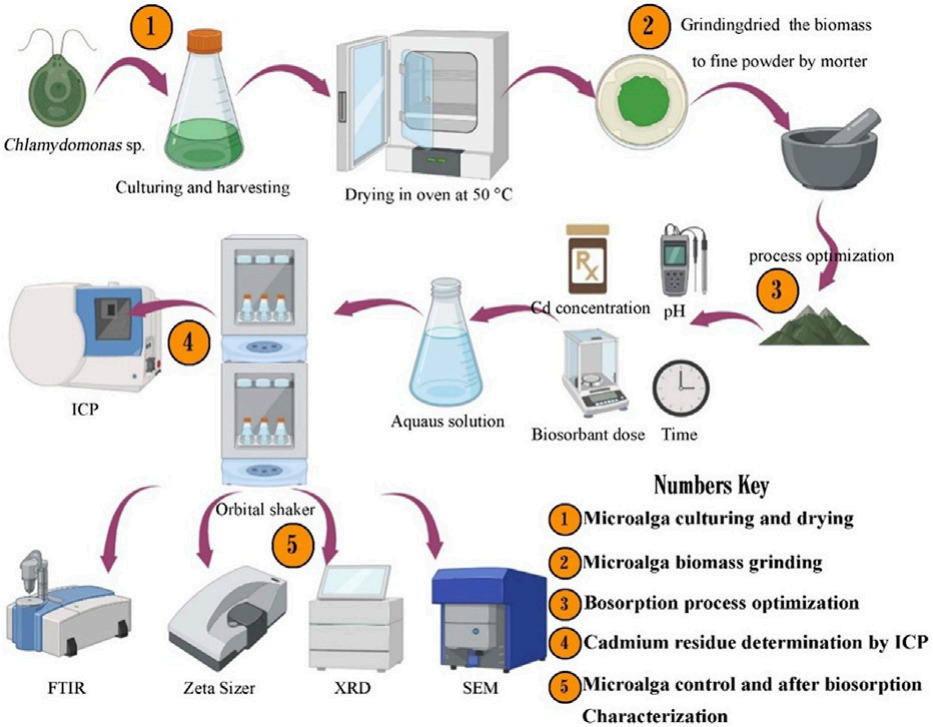
Illustrates the pathway for preparing biosorbent, performing biosorption experiments, and conducting biomass characterization.

In this study, the biosorbent microalga *Chlamydomonas* sp. was kindly provided by the Algal Biotechnology Lab, Botany and Microbiology Department, Faculty of Science, Beni-Suef University, Egypt, followed by re-subculture in enrichment growth medium (BG11), state of light 12 h light/12 h dark cycle (photon flux density: 50 μmol m^−2^ s^−1^), temperature of 25°C ± 1°C, and constant aeration through filtered air (0.22 µm filter), to obtain an intensive biomass production sufficient for metal binding experiments (Rippka et al., 1979). The harvested biomass underwent drying in an oven set at 50°C until complete desiccation was achieved. Subsequently, the dried biomass was meticulously ground to yield a fine powder with uniform particle size, thereby enhancing its biosorption efficacy as demonstrated in (Figure 1) (Kashyap et al., 2019).

### 2.2 Preparation of cadmium solution

A stock solution of cadmium sulfate (Sigma-Aldrich), exhibiting a purity of at least 99.0% and characterized by its solubility in water, was formulated following the methodology articulated by Alpat et al. (2010). In detail, a concentration of 1 g/L of cadmium sulfate (3CdSO_4_·8H_2_O) was dissolved in distilled water to formulate a solution appropriate for conducting adsorption experiments.

### 2.3 Optimization of the adsorption process

Adsorption experiments were conducted employing dried *Chlamydomonas* biomass under various conditions to optimize the process. The optimization of this adsorption process required a comprehensive evaluation of several parameters to increase efficiency. Adsorption experiments were carried out in 50 mL of aqueous solution, utilizing 0.1 M HCl and NaOH to adjust the solution pH values to 3, 4, 5, 6, 7, and 8, in order to determine their effect on adsorption efficiency while preventing precipitation (Ibrahim et al., 2016). The effect of biosorbent dosage was analyzed by altering the quantity of microalgal biomass to 0.2, 0.4, 0.6, 0.8, 1.0, and 1.2 g/L (Kahraman et al., 2005). Cadmium concentrations of 25, 50, 100, 140, 180, and 200 mg/L were utilized to examine the equilibrium adsorption process (Kumar et al., 2020). Furthermore, the process underwent examination under variable temperature conditions of 15, 20, 25, 30, 35, and 40 °C to evaluate their influence on adsorption kinetics and thermodynamics. Additionally, the effect of contact time was monitored across several durations, ranging from 10 to 160 min, to ascertain the equilibrium time. Centrifugation was conducted at 3,000 rpm for 15 min to separate biomass from the aqueous solution upon conclusion of the experiment (Alpat et al., 2010).

### 2.4 Adsorption modeling and analysis

The adsorption process was evaluated through the utilization of diverse models to determine its efficiency, capacity, kinetics, isotherms, thermodynamics, and the feasibility of biosorbent regeneration. The mathematical models used in the analysis are summarized in Table 1. The efficacy of cadmium removal and adsorption capacity was determined through the application of fundamental equations for R% and qe. A range of kinetic models, encompassing pseudo-first-order, pseudo-second-order, Elovich, and intraparticle diffusion, were employed to elucidate the temporal characteristics of the adsorption process. Isotherm models, including Langmuir and Freundlich, were utilized to characterize the equilibrium adsorption behavior (Umpleby et al., 2001), while the Temkin and Dubinin–Radushkevich models provided critical insights into adsorbate–adsorbent interactions and adsorption energy, respectively (Foo and Hameed, 2010). Thermodynamic assessments were conducted to evaluate the feasibility and spontaneity of the adsorption process, whereas the regeneration of the biosorbent was analyzed through equations pertaining to desorption efficiency and capacity.

**TABLE 1:** Equations used in adsorption modeling and analysis.

Category	Equation	Description
Adsorption Efficiency	R% = ((Ci - Ce)/Ci) × 100	R%: Cadmium removal efficiency; Ci: Initial concentration; Ce: Equilibrium concentration
Adsorption Capacity	qe = ((Ci - Ce) × V)/M	qe: Adsorption capacity (mg/g); V: Solution volume (L); M: Biosorbent mass (g)
Kinetics: Pseudo-First-Order	ln (qe - qt) = ln (qe) - K1t	qt: Adsorption at time t; K1: Rate constant
Kinetics: Pseudo-Second-Order	t/qt = t/qe + 1/(K2qe^2^)	K2: Rate constant for second-order kinetics
Kinetics: Elovich Model	qt = b ln (ab) + b ln(t)	b: Activation energy; a: Initial rate; t: Contact time
Kinetics: Intraparticle Diffusion	qt = Kdiff t^0.5^ + C	Kdiff: Diffusion rate constant; C: Intercept for boundary layer effect
Langmuir Isotherm	Ce/qe = (1/qmaxb) + (Ce/qmax)	Monolayer adsorption model; qmax: Maximum adsorption capacity; b: Langmuir constant
Freundlich Isotherm	log (qe) = log (Kf) + (1/n) log (Ce)	Multilayer adsorption model; Kf: Freundlich constant; n: Adsorption intensity
Temkin Isotherm	qe = B ln (AT) + B ln (Ce), B = RT/bT	Adsorbate–adsorbent interaction model; B: Adsorption heat constant; AT: Binding constant
Dubinin–Radushkevich Isotherm	ln (qe) = ln (qm) - Kε^2^, ε = RT ln (1 + 1/Ce)	Energy and porosity model; qm: Maximum adsorption capacity; K: D–R constant
Gibbs Free Energy	ΔG° = -RT ln(K)	Thermodynamic parameter for spontaneity; K: Equilibrium constant
Enthalpy and Entropy	ln(K) = (ΔS°/R) - (ΔH°/RT)	Thermodynamic parameters for adsorption heat and disorder; R: Gas constant; T: Temperature
Desorption Efficiency	E% = (Cde/Cad) × 100	Desorption efficiency; Cde: Desorbed cadmium; Cad: Adsorbed cadmium
Removal Percentage	Removal% = ((Ci - Ct)/Ci) × 100	Percentage of cadmium removed; Ct: Concentration at time t
Desorption Capacity	qde = (Cde × V)/M	Desorption capacity (mg/g); V: Eluent volume (L)

### 2.5 Biomass characterization

The characterization of the biosorbent was conducted using a comprehensive array of analytical methodologies. Fourier Transform Infrared Spectroscopy (FTIR) analysis, performed with a Bruker Vertex 70 spectrometer (OPUS version 7.2, United States), enabled the identification of functional groups present on the biomass surface by capturing spectra at a resolution of 4 cm^-1^ across the range of 4,000–400 cm^-1^. The crystalline architecture of the biosorbent was examined via X-ray diffraction (XRD) utilizing a Rigaku MiniFlex II diffractometer, over a 2θ angular span of 10°–90°. Scanning Electron Microscopy (SEM - EDX) using a JSM-6510LA Series (Japan) provided detailed insights into the morphological attributes and elemental composition of the biomass pre- and post-adsorption. The particle size distribution and uniformity of the dry biomass were assessed through Dynamic Light Scattering (DLS), while the zeta potential, determined using a Malvern Zeta Sizer (United Kingdom), illuminated the influence of surface charge on pH and adsorption capacity. Subsequently, the cadmium concentration in the supernatant was measured using Inductively Coupled Plasma (ICP) analysis (PerkinElmer Avio 220 Max, United States), with results presented as mean values accompanied by standard deviations.

### 2.6 Regeneration and reuse

In order to assess the reusability of dried *Chlamydomonas* biomass as a biosorbent, a range of eluent agents, HNO_3_, H_2_SO_4_, HCl, 0.1 M NaOH, and EDTA, were evaluated for their efficiency in desorbing cadmium ions under meticulously controlled conditions (cadmium concentration of 25 mg/L, biomass dose of 0.8 g/L, temperature 25°C, pH 4, and contact time of 60 min). Post-desorption, the supernatant was subjected to analysis via atomic absorption spectroscopy to ascertain the desorption efficiency. To determine the optimal contact time, the biosorbent was subjected to the eluent agents for durations ranging between 10 and 90 min, thereby identifying the optimal period for maximum cadmium recovery. EDTA, identified as the most efficacious eluent agent, was employed to evaluate the biosorbent’s regeneration potential through five successive adsorption-desorption cycles. Between cycles, the biosorbent underwent washing with deionized water, drying at 60°C for 24 h, and reuse under consistent conditions. The adsorption and desorption efficiencies were systematically recorded after each cycle to determine the biosorbent’s durability and reusability.

### 2.7 Statistical analysis

All experiments were conducted in triplicate, and results were expressed as mean ± standard deviation (SD). Statistical analysis was performed using Microsoft Excel 2019 and Origin software.

## 3 Results

### 3.1 Optimization of biosorption conditions

#### 3.1.1 Effect of pH on adsorption capacity

**FIGURE 2:**
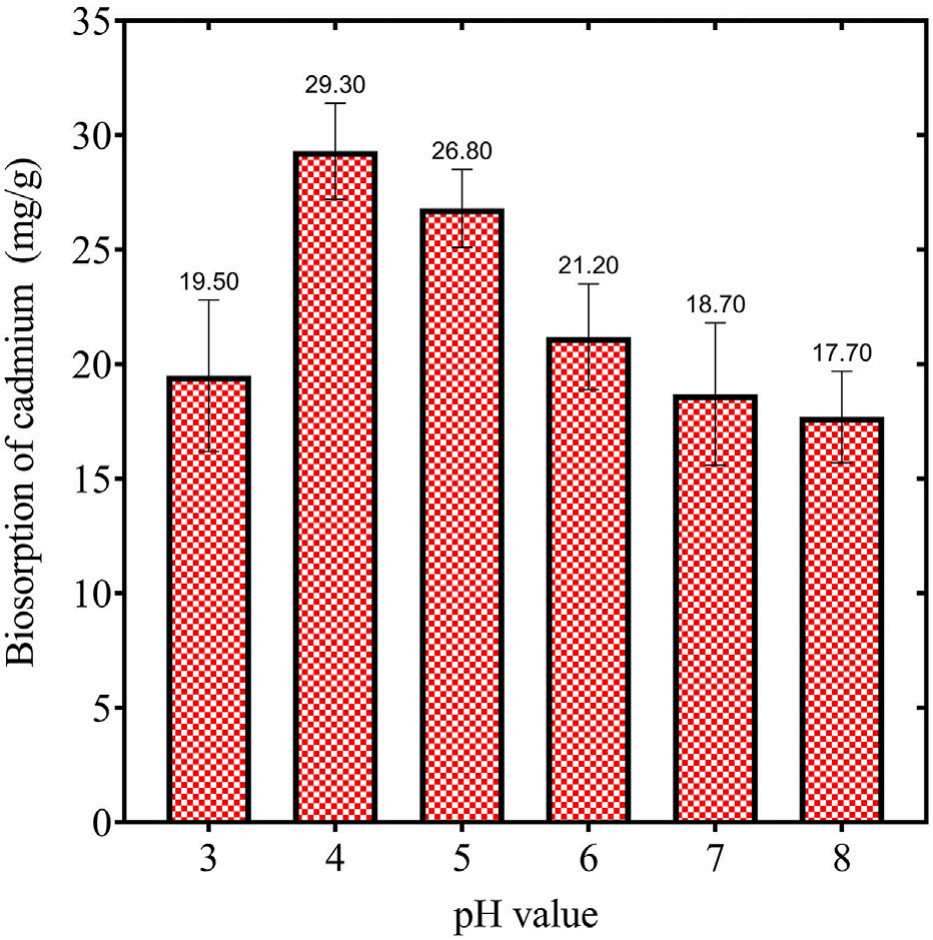
Illustrates the effect of pH on the cadmium biosorption by dried *Chlamydomonas* microalgae, maintained under constant conditions of time (60 min), biosorbent dose (1 gm/L), temperature (25°C), and heavy metal concentration (50 mg/L).

As illustrated in (Figure 2), at pH 3, the biosorption capacity was approximately 19.50 mg/g, indicating a moderate level of uptake under highly acidic conditions. A significant increase in biosorption capacity was observed as the pH was elevated to 4, achieving maximum adsorption of 29.30 mg/g. This finding suggests that pH 4 represents the optimal condition for the adsorption of cadmium by the biosorbent. Subsequently, the capacity decreased slightly to 26.80 mg/g at pH 5, implying a reduction in adsorption efficiency as the solution becomes less acidic. Beyond pH 5, the biosorption capacity decreased progressively. At pH 6, the adsorption diminished to 21.20 mg/g, and at pH 7 and pH 8, the values were 18.70 mg/g and 17.70 mg/g, respectively. The observed decline in biosorption at elevated pH values may be attributed to the precipitation of cadmium ions as insoluble cadmium hydroxides, coupled with reduced availability of active binding sites due to alterations in surface charge. These findings indicate that acidic conditions, particularly at pH 4, are conducive to cadmium biosorption. The trend observed suggests that both the surface charge of the biosorbent and the ionic state of cadmium ions are critical determinants of the adsorption process. The peak at pH 4 is consistent with the pKa values of functional groups on the biosorbent surface, thereby facilitating stronger binding interactions between cadmium ions and the biosorbent. As pH rises beyond the optimal level, competition between hydroxyl ions and cadmium ions for active sites may result in diminished adsorption efficiency.

**FIGURE 3:**
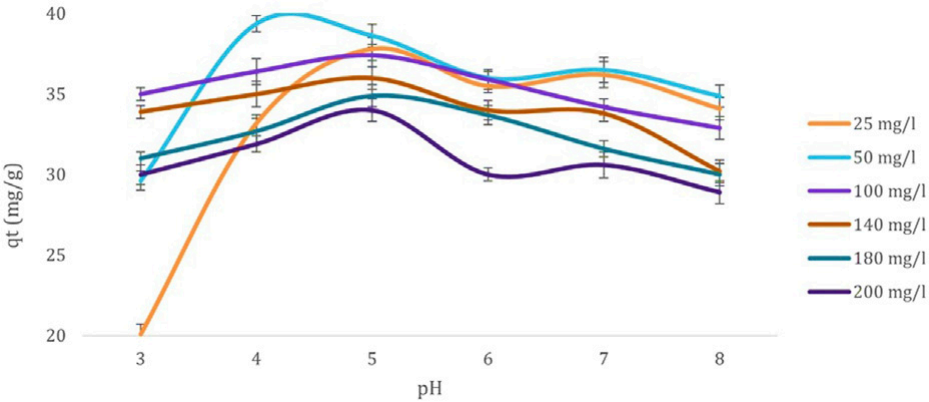
Effect of pH (3, 4, 5, 6, 7, and 8) on the biosorption capacity (mg/g) of Cd^2+^ ions at various initial concentrations (25, 50, 100, 140, 180, and 200 mg/L) using dried *Chlamydomonas* biomass. Experimental conditions: temperature 25°C, contact time 60 min, and biosorbent concentration 0.8 g/mL.

Further, Figure 3 illustrates varying pH levels’ influence on cadmium ions’ biosorption capacity by dried *Chlamydomonas* biomass. The adsorption capacity increased steadily with rising pH, peaking at 29.3 mg/g at pH 4. This trend reflects the increasing negative charge on the biosorbent surface, which enhances the attraction of positively charged cadmium ions. As the pH increased to 4, the reduction in competing hydrogen ions facilitated greater adsorption. Beyond this point, further pH increases to 7 led to a gradual decline in adsorption capacity due to the formation of insoluble cadmium hydroxide. The interaction between OH^−^ anions and cadmium cations likely contributed to metal precipitation. High concentrations of H^+^ ions compete with cadmium cations at lower pH levels for binding sites on the biosorbent, resulting in reduced adsorption. The optimal adsorption observed at pH 4 indicates a balance where competition from H^+^ ions is minimized, and cadmium ions can effectively bind to active sites. Beyond pH 4, the emergence of OH^−^ ions introduces competition that slightly reduces cadmium adsorption, with a steady decline in performance up to pH 7. At pH 8, the adsorption capacity stabilizes under slightly alkaline conditions. This behavior emphasizes the role of pH in modulating the biosorption efficiency of heavy metal ions.

#### 3.1.2 Effect of biomass dose on adsorption capacity

**FIGURE 4:**
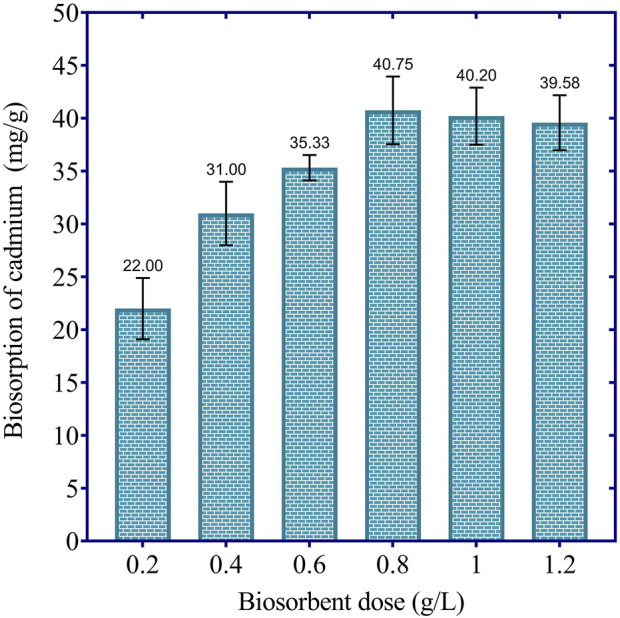
Effect of biosorbent dosage on cadmium biosorption efficiency by dried *Chlamydomonas* at 60 min, pH 4, temperature 25°C, and initial cadmium concentration of 50 mg/L.

The results in Figure 4 illustrate the effect of biosorbent dosage on the cadmium removal efficiency of dried *Chlamydomonas*. At the lowest biosorbent dosage of 0.2 g/L, the biosorption capacity was 22.00 mg/g, corresponding to a removal efficiency of 44% of the initial cadmium load. Increasing the dosage to 0.4 g/L enhanced the biosorption capacity to 31.00 mg/g, achieving a 62% removal efficiency. Further increasing the dosage to 0.6 g/L resulted in a biosorption capacity of 35.33 mg/g, which corresponds to a removal efficiency of 70.66%. The highest cadmium removal was observed at 0.8 g/L, with a biosorption capacity of 40.75 mg/g, achieving an 81.5% removal efficiency. However, beyond this optimal dosage, the removal efficiency began to stabilize or slightly decline, with biosorption capacities of 40.20 mg/g (80.4% removal) at 1.0 g/L and 39.58 mg/g (79.16% removal) at 1.2 g/L. This slight decline is likely due to particle aggregation at higher biosorbent concentrations, which reduces the effective surface area and limits the availability of active binding sites. These results suggest that a biosorbent dosage of 0.8 g/L is optimal for maximizing cadmium removal under the given conditions, achieving over 81% removal efficiency while avoiding excessive biosorbent use and its associated diminishing returns.

#### 3.1.3 Effect of initial Cd^2+^ concentration on biosorption efficiency and equilibrium time

**FIGURE 5:**
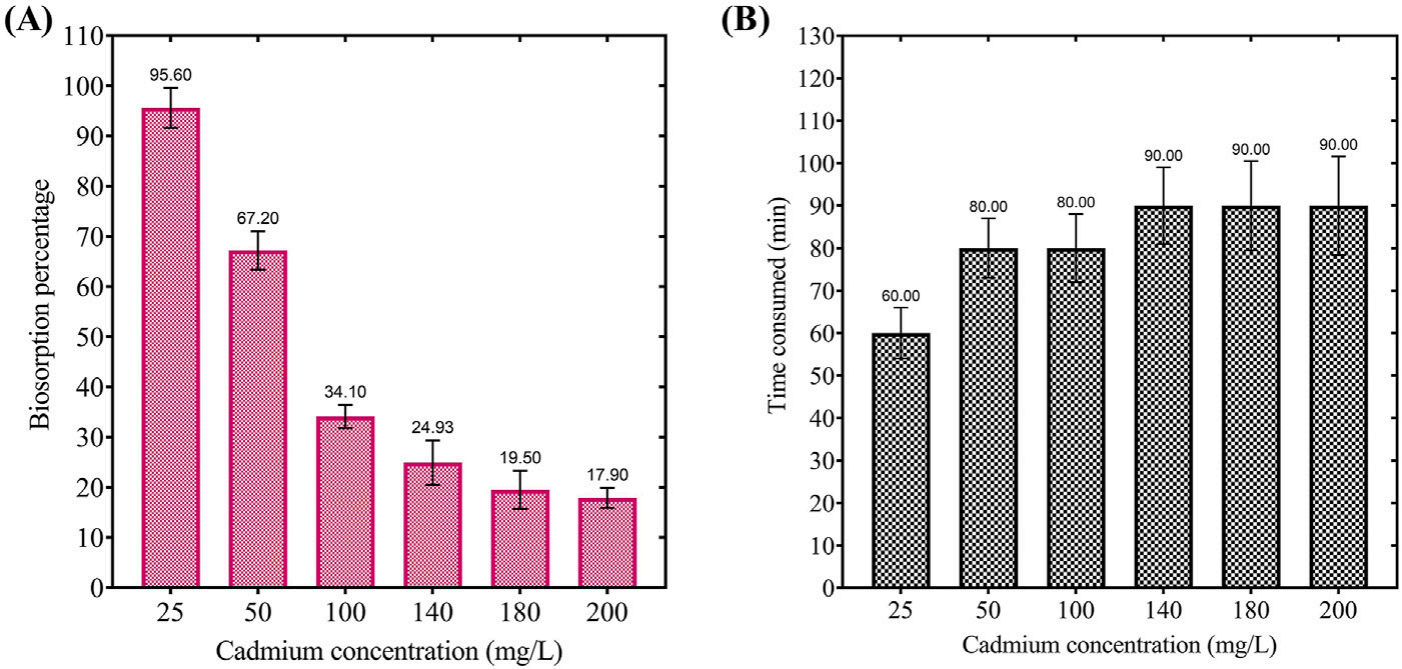
**(A)** Effect of initial cadmium concentration on the biosorption percentage by dried *Chlamydomonas* at pH 4, temperature 25°C, biosorbent dosage of 0.8 g/mL, and contact time of 60 min. **(B)** Effect of initial cadmium concentration on the equilibrium time required for cadmium biosorption under the same conditions.

The results presented in Figure 5 highlight the impact of initial cadmium concentration on the biosorption efficiency and equilibrium time for cadmium removal. Panel A demonstrates a significant decline in biosorption efficiency as the initial cadmium concentration increases, with a removal percentage of 95.60% at 25 mg/L, decreasing to 67.20% at 50 mg/L, and further to 34.10%, 24.93%, 19.50%, and 17.90% at 100 mg/L, 140 mg/L, 180 mg/L, and 200 mg/L, respectively. This trend indicates that at higher cadmium concentrations, the binding sites on the biosorbent become saturated, limiting its removal efficiency. Further, Panel B reveals that equilibrium time increases with initial cadmium concentration, requiring 60 min at 25 mg/L, 80 min at 50 mg/L and 100 mg/L, and 90 min at 140 mg/L and above, reflecting the greater ion competition for active binding sites at higher concentrations. These findings suggest that the biosorbent exhibits high efficiency and rapid equilibrium at lower cadmium concentrations. In comparison, at higher concentrations, efficiency is constrained by site saturation and prolonged equilibrium times, emphasizing the importance of optimizing initial concentrations for bioremediation practical applications.

#### 3.1.4 Effect of temperature on biosorption

**FIGURE 6:**
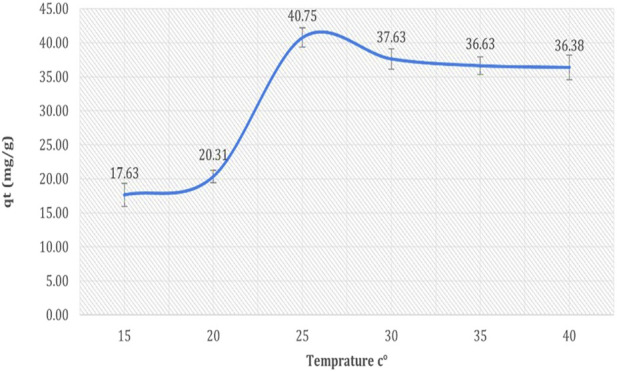
Effect of temperature on biosorption of cadmium by dried *Chlamydomonas* at pH 4, contact time of 60 min, biosorbent dose of 0.8 g/mL, and heavy metal concentration of 50 mg/L.

The results presented in Figure 6 demonstrate the influence of temperature on the biosorption capacity of cadmium by *Chlamydomonas*, revealing an optimal temperature for maximum biosorption efficiency. At 15°C, the biosorption capacity was 17.63 mg/g, corresponding to a cadmium removal efficiency of 35.26%. With an increase in temperature to 20°C, the biosorption capacity improved to 20.31 mg/g, achieving a removal efficiency of 40.62%. The highest biosorption capacity, 40.75 mg/g, was recorded at 25°C, indicating a maximum removal efficiency of 81.50%. Beyond 25°C, the biosorption capacity began to decrease slightly, with values of 37.63 mg/g, 36.63 mg/g, and 36.38 mg/g observed at 30°C, 35°C, and 40°C, corresponding to removal efficiencies of 75.26%, 73.26%, and 72.76%, respectively. These results suggest that the biosorption process is temperature-dependent, with a significant enhancement in biosorption efficiency as the temperature increases to 25°C, likely due to improved molecular motion and increased interaction between cadmium ions and the biosorbent’s active sites. However, the slight decline in biosorption capacity at temperatures above 25°C may be attributed to desorption effects or disruption of binding interactions caused by thermal agitation.

### 3.2 Adsorption kinetics

**FIGURE 7:**
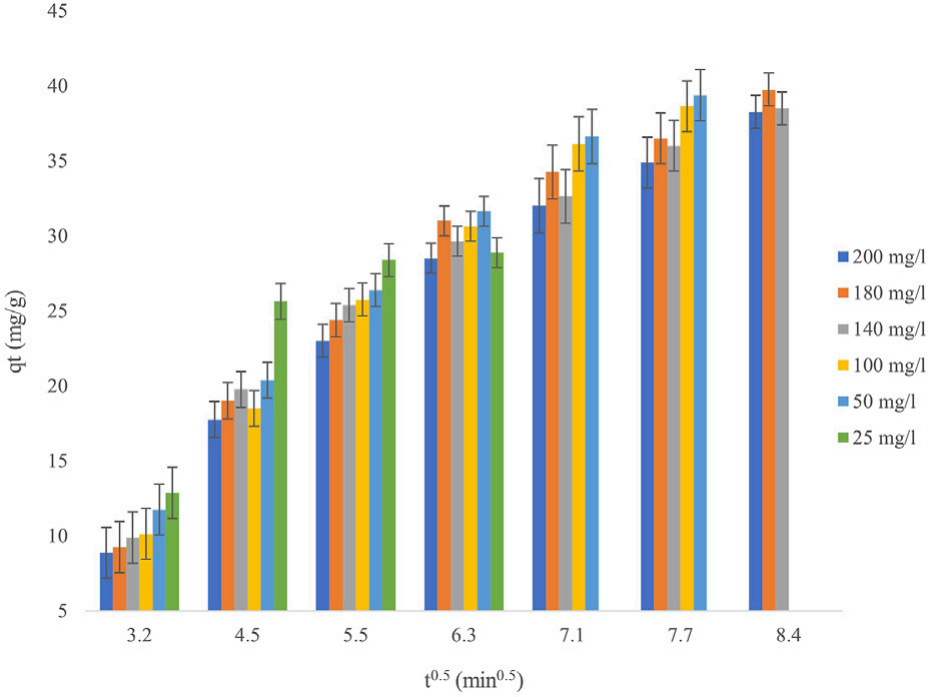
Intraparticle diffusion kinetics for Cd^2+^ biosorption at initial heavy metal concentrations ranging from 25 to 200 mg/L, using a biosorbent dose of 0.8 g/L, at pH 4 and a temperature of 25°C.

The kinetics of cadmium adsorption onto the biosorbent were evaluated using pseudo-first-order (PFO), pseudo-second-order (PSO), Elovich, and intraparticle diffusion (IPD) models, as illustrated in Figure 7, utilizing the equations provided in Table 1. The findings reveal a multi-phase adsorption process, with the PSO model exhibiting the optimal fit (R^2^ > 0.99) at all concentrations, hence affirming chemisorption as the prevailing mechanism. The PFO model produced rate constants (k_1_) ranging from 0.012 to 0.025 min^–1^, while equilibrium adsorption capacities (q_e) rose from 34.2 mg/g at 25 mg/L Cd^2+^ to over 40 mg/g at elevated starting concentrations (180–200 mg/L). Nonetheless, the reduced correlation coefficients (R^2^ = 0.82–0.89) at increased Cd^2+^ concentrations indicate that physical adsorption is not the predominant mechanism. The IPD model, exhibits a multi-linear trend, signifying separate adsorption phases. The initial quick phase (k_id_1_ = 2.1–3.8 mg g^−1^ min^−0.5^) pertains to surface adsorption, whereas the following slower phase (k_id_2_ = 0.4–0.9 mg g^−1^ min^−0.5^) indicates constraints due to pore diffusion. The existence of non-zero intercepts (C = 6.2–9.8 mg/g) validates boundary layer effects, emphasizing that intraparticle diffusion is not the only rate-limiting factor. The Elovich model corroborates these findings, indicating an ascending initial adsorption rate (α) from 8.5 to 12.4 mg g^−1^ min^−1^ as Cd^2+^ concentration increases (25–200 mg/L), whereas the desorption constant (β) diminishes from 0.18 to 0.11 g/mg. These patterns indicate increased adsorption at elevated Cd^2+^ concentrations owing to intensified driving forces, coupled with a heterogeneous biosorbent surface, akin to walnut shell-based adsorbents (α = 10.2–14.7 mg g^−1^ min^−1^). Similar multi-phase diffusion characteristics have been seen in lignocellulosic biosorbents, with surface adsorption prevailing in the earliest phases. The adsorption capacity of the biosorbent corresponds with documented values for algae-derived materials; however, the measured kinetics (k_2_ = 10^−3^–10^−4^ g mg^−1^ min^−1^) are diminished at elevated Cd^2+^ concentrations, presumably due to constraints on active sites. The Elovich parameters further emphasize a heterogeneous surface, aligning with the variety of functional groups present in biomass-derived adsorbents. Kinetic research indicates that chemisorption controls cadmium uptake, while intraparticle diffusion plays a role in the later phases. The decrease in k_2_ values and extended equilibration at elevated Cd^2+^ concentrations (180–200 mg/L) suggest competition binding for active sites, a well-established occurrence in biosorbent systems. These findings highlight the biosorbent’s efficacy for low-to-moderate cadmium concentrations, demonstrating performance akin to those of conventional biosorbents.

### 3.3 Adsorption isotherm

**FIGURE 8:**
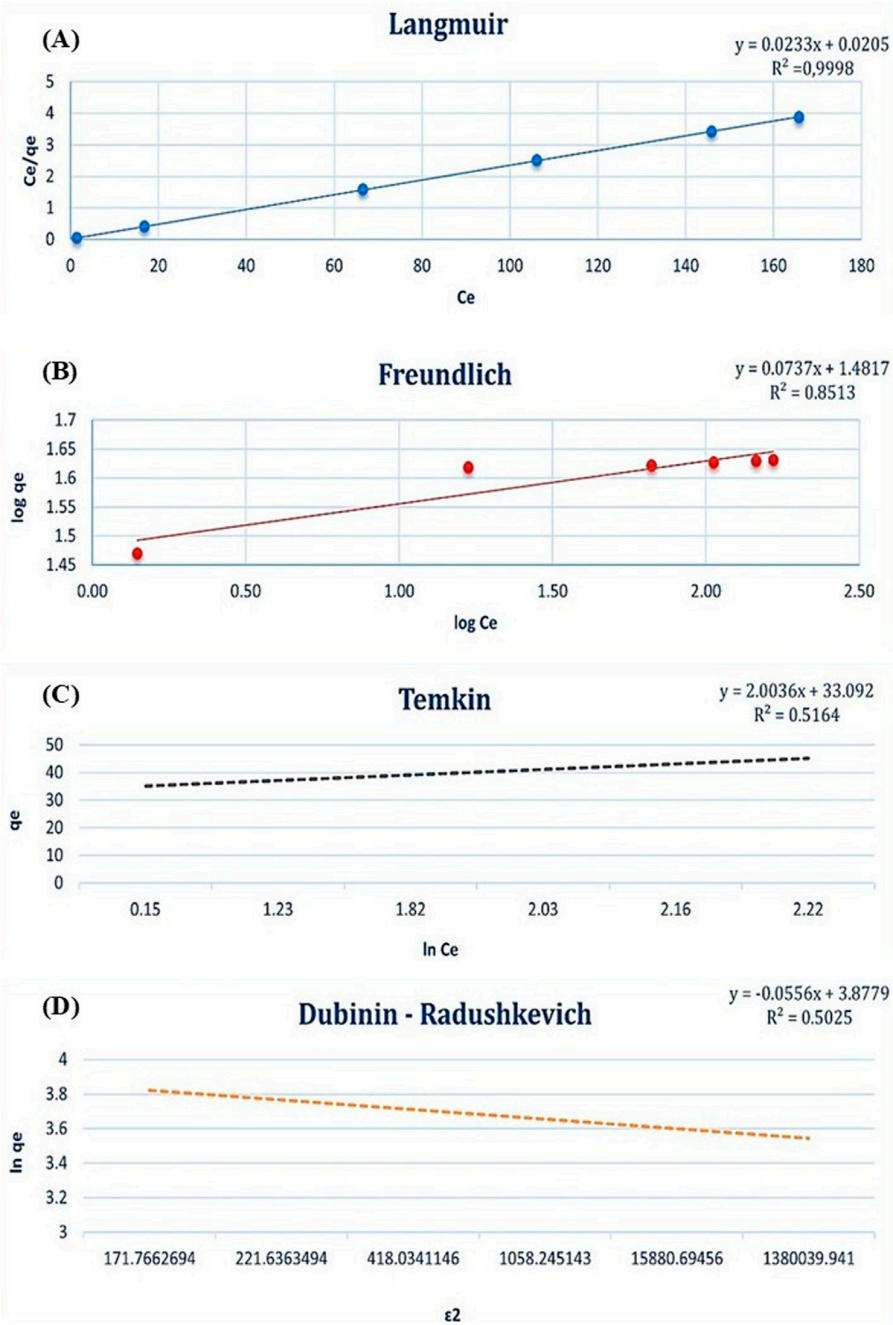
Adsorption isotherm models for Cd^2+^ biosorption: **(A)** Langmuir isotherm, **(B)** Freundlich isotherm, **(C)** Temkin isotherm, and **(D)** Dubinin-Radushkevich isotherm.

According to the comparative analysis of adsorption isotherm models (Dubinin-Radushkevich, Langmuir, Freundlich, and Temkin), the Langmuir isotherm displayed the highest R2 value of 0.9998 for the Cd^2+^ adsorption model, as shown in Figure 8. In contrast, the R2 values for the Freundlich, Temkin, and Dubinin-Radushkevich models were significantly lower, at 0.8513, 0.5164, and 0.5025, respectively. These results indicate that the Langmuir isotherm provides the best fit for describing the adsorption process of Cd^2+^ ions, suggesting that the adsorption occurs on a homogenous surface with constant energy for adsorption. This implies that the interaction between the adsorbent and adsorbate molecules is uniform and stronger than in the other models analyzed. The Langmuir model’s high R2 value supports the hypothesis that adsorption follows a monolayer mechanism, where the first molecule adsorbed at a binding site facilitates the adsorption of subsequent molecules through consistent and strong adsorbent-adsorbate interaction. However, the strength of this interaction diminishes as additional molecules are adsorbed. Furthermore, the uniformity of the adsorption surface increases the likelihood of active binding sites being present on the dried biomass, reinforcing the consistency of the Langmuir isotherm. The maximum adsorption capacity for Cd^2+^ was determined to be 42.9 mg/g, attributable to the homogeneity of the adsorbent surface. These findings underscore the efficiency of the Langmuir model in characterizing the adsorption process and highlight the importance of surface uniformity in achieving high adsorption capacities.

### 3.4 Adsorption thermodynamics

**FIGURE 9:**
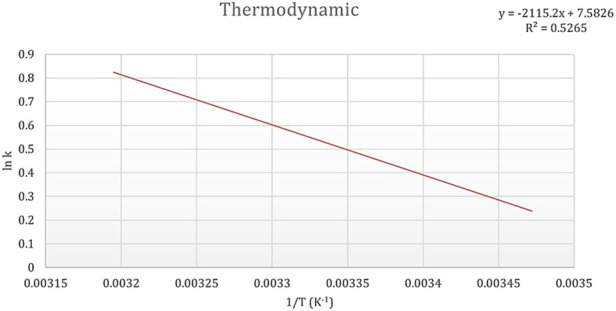
Thermodynamic graph illustrating the effect of temperature (15°C, 20°C, 25°C, 30°C, 35°C, and 40°C) on the biosorption of cadmium by dried *Chlamydomonas* sp. microalgae under controlled conditions (pH 4, contact time of 60 min, biosorbent dose of 0.8 g/mL, and cadmium concentration of 50 mg/L).

The ability of dried microalgae to remove heavy metals was evaluated across a temperature range of 15°C, 20°C, 25°C, 30°C, 35°C, and 40°C, as shown in Figure 9. The findings indicate that the bioremoval process is thermally dependent, with an optimal temperature of 25°C identified for maximum efficiency. At the tested temperatures, the calculated Gibbs free energy (ΔG) values were negative, measuring −0.3034 kJ/mol, −0.4591 kJ/mol, −2.1084 kJ/mol, −1.8056 kJ/mol, and −1.7375 kJ/mol, respectively, highlighting the spontaneity of the process under these conditions. The lowest free energy at 25°C aligns with the optimal temperature for heavy metal removal. Additionally, the positive enthalpy (ΔH) value of +17.58 kJ/mol confirms that the bioremoval process is endothermic, suggesting that heat enhances the interaction between heavy metal ions and the microalgae biosorbent. The entropy (ΔS) value of +63.04 J/mol·K indicates an increase in randomness during the adsorption process, which further supports the spontaneous binding of Cd^2+^ ions to the biosorbent. The positive entropy change suggests a strong attraction between the Cd^2+^ ions and the active sites on the algal biomass.

### 3.5 Inorganic ions effects

**FIGURE 10:**
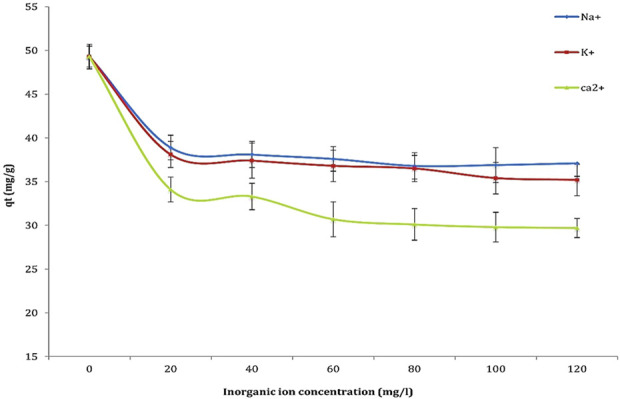
Investigation of the effect of inorganic ions (Na^+^, K^+^, and Ca^2+^) on the biosorption capacity of Cd^2+^ under controlled conditions: Cd^2+^ concentration of 50 mg/L, inorganic ion concentrations ranging from 20 to 120 mg/L, contact time of 60 min, pH 4, temperature 25°C, and biosorbent dose of 0.8 g/mL.

The effect of surrounding inorganic ions on the interaction between the sorbent and sorbate was investigated to assess their influence on the adsorption of Cd^2+^ ions. The impact of Na^+^, K^+^, and Ca^2+^ ions on cadmium biosorption was examined, and the results are presented in Figure 10. The findings indicate that monovalent ions (Na^+^ and K^+^) exert a negligible effect on the adsorption process, even at elevated concentrations. Conversely, divalent ions (Ca^2+^) demonstrated a more pronounced inhibitory effect, with the order of inhibition observed as Ca^2+^ > K^+^ > Na^+^. This suggests that divalent ions, due to their stronger binding affinity for active sites on the biosorbent surface, are more likely to compete with Cd^2+^ ions for these sites. The competition introduced by Na^+^, K^+^, and Ca^2+^ ions reduces the availability of active adsorption sites for Cd, leading to a decrease in the overall metal uptake. This competition impairs cadmium transport to the biosorbent surface, as these ions occupy active binding sites. Furthermore, the presence of divalent ions, particularly Ca^2+^, promotes the aggregation of organic ions through the compression of the electric double layer on the biosorbent surface, further hindering the adsorption of Cd^2+^. The stronger inhibitory effect of Ca^2+^ compared to Na^+^ and K^+^ can be attributed to its higher affinity for hydroxyl groups on the biosorbent surface. These results emphasize the critical need to account for the presence of competing ions, especially divalent ions like Ca^2+^ when designing and optimizing biosorption processes for the removal of heavy metals.

### 3.6 Biomass characterization

#### 3.6.1 FTIR spectrum

**FIGURE 11:**
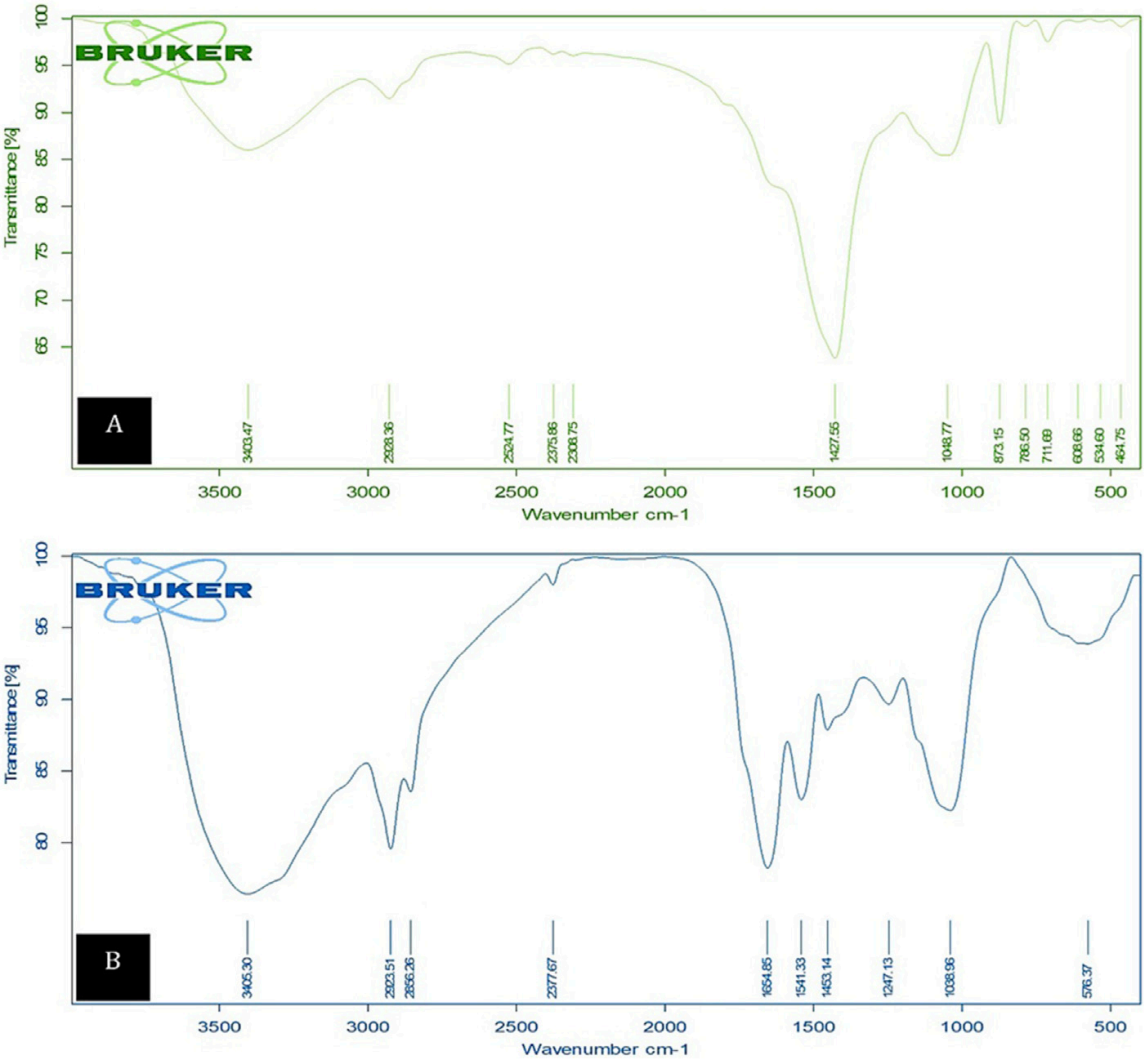
FTIR spectra showing characteristic peaks for **(A)** unloaded control biomass and **(B)** Cd^2+^-loaded control biomass, highlighting the functional groups involved in the biosorption process.

The FTIR spectral analysis of the biomass was conducted to identify the functional groups responsible for Cd^2+^ binding during the adsorption process. As depicted in Figure 11, the spectra were recorded within the range of 400–4,000 cm^−1^, both before and after adsorption. Before the adsorption process, the FTIR spectrum of the dried microalgae revealed prominent peaks at 3,403.74, 2,928.36, 2,524.77, 2,375.86, 2,308.75, 1,427.56, 1,048.77, 873.15, 786.50, 608.66, 534.60, and 464.75 cm^−1^. Notably, following cadmium adsorption, these peaks underwent significant transformations, including broadening, merging, and a shift to 3,422 cm^−1^, suggesting structural and compositional changes in the biomass. The FTIR analysis further revealed that the microalgal biomass contained a range of vibrational frequencies corresponding to diverse functional groups. A broad band observed at 3,422.94 cm^−1^ indicated the presence of hydroxyl (OH) groups, both hydrogen-bonded and non-hydrogen-bonded. The protein amide groups (C-N and N-H stretching) were identified at 1,422.34 cm^−1^, while carbonyl group stretching (-HC = O, R2C = O) was detected at 1,640.47 cm^−1^. The aliphatic C-H stretching vibration appeared at 2,928.36 cm^−1^, and the S-H stretching characteristic of Chlamydomonas was observed at 2,376.00 cm^−1^ and 1,422.34 cm^−1^. Additionally, asymmetric stretching of Si-O-Si bonds and free C=O groups was detected at 1,047.26 cm^−1^, while metal compounds were represented by bands at 801.28 cm^−1^. The analysis confirms that the microalgae biomass comprises carbohydrates, lipids, and proteins, which serve as the primary structural components containing active and adaptable functional groups. The most significant changes in the biomass were observed in the 1,000–1,600 cm^−1^ range, which is associated with carboxyl, amino, and hydroxyl groups. The increased bond intensity at 3,400 cm^−1^ in the O-H stretching region, as demonstrated in the Cd^2+^-native and Cd^2+^-loaded FTIR spectra of the algal biomass, confirms the involvement of these functional groups in the binding of heavy metal cations. This comprehensive analysis highlights the role of active groups, such as hydroxyl, carboxyl, and amino groups, in cadmium adsorption by Chlamydomonas biomass.

#### 3.6.2 X-ray diffraction

**FIGURE 12:**
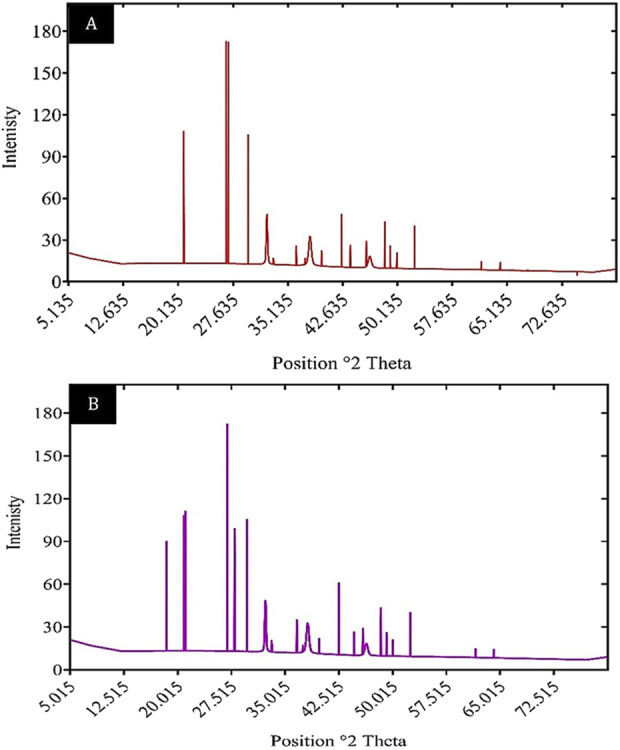
XRD patterns of **(A)** unloaded biomass and **(B)** Cd^2+^ loaded biomass, illustrating changes in crystalline structure.

X-ray diffraction (XRD) is a well-established technique for analyzing the crystalline properties and structural arrangement of materials. It is frequently utilized to characterize biosorbents and confirm the adsorption of heavy metals. As illustrated in Figure 12, the XRD patterns of unloaded and Cd^2+^-loaded biomass highlight significant changes in the crystalline structure following the adsorption process. The unloaded biosorbent displayed distinct peaks at 20.8837°, 26.6952°, and 29.6743°, with corresponding d-spacing values of 4.25374 Å, 3.33944 Å, and 3.01062 Å, respectively. In comparison, the XRD pattern of Cd^2+^-loaded biomass revealed the appearance of additional peaks, including a notable peak at approximately 13.804°, indicating structural modifications due to cadmium binding.

The XRD results for the unloaded biosorbent reflect sharp diffraction peaks, characteristic of the crystalline nature of the *Chlamydomonas* biomass. However, after Cd^2+^ adsorption, changes in the 2-theta angles and d-spacing values suggest alterations in the biosorbent’s crystalline structure. These modifications are attributed to the interaction between the Cd^2+^ ions and the biosorbent, which leads to rearrangements within the crystal lattice. The appearance of new peaks in the Cd^2+^-loaded biosorbent confirms that the material effectively adsorbs metal ions, further validating its biosorption capability. These findings emphasize the utility of XRD in elucidating structural changes in biosorbents during heavy metal adsorption and provide valuable insights into the underlying adsorption mechanisms.

#### 3.6.3 SEM technology

**FIGURE 13:**
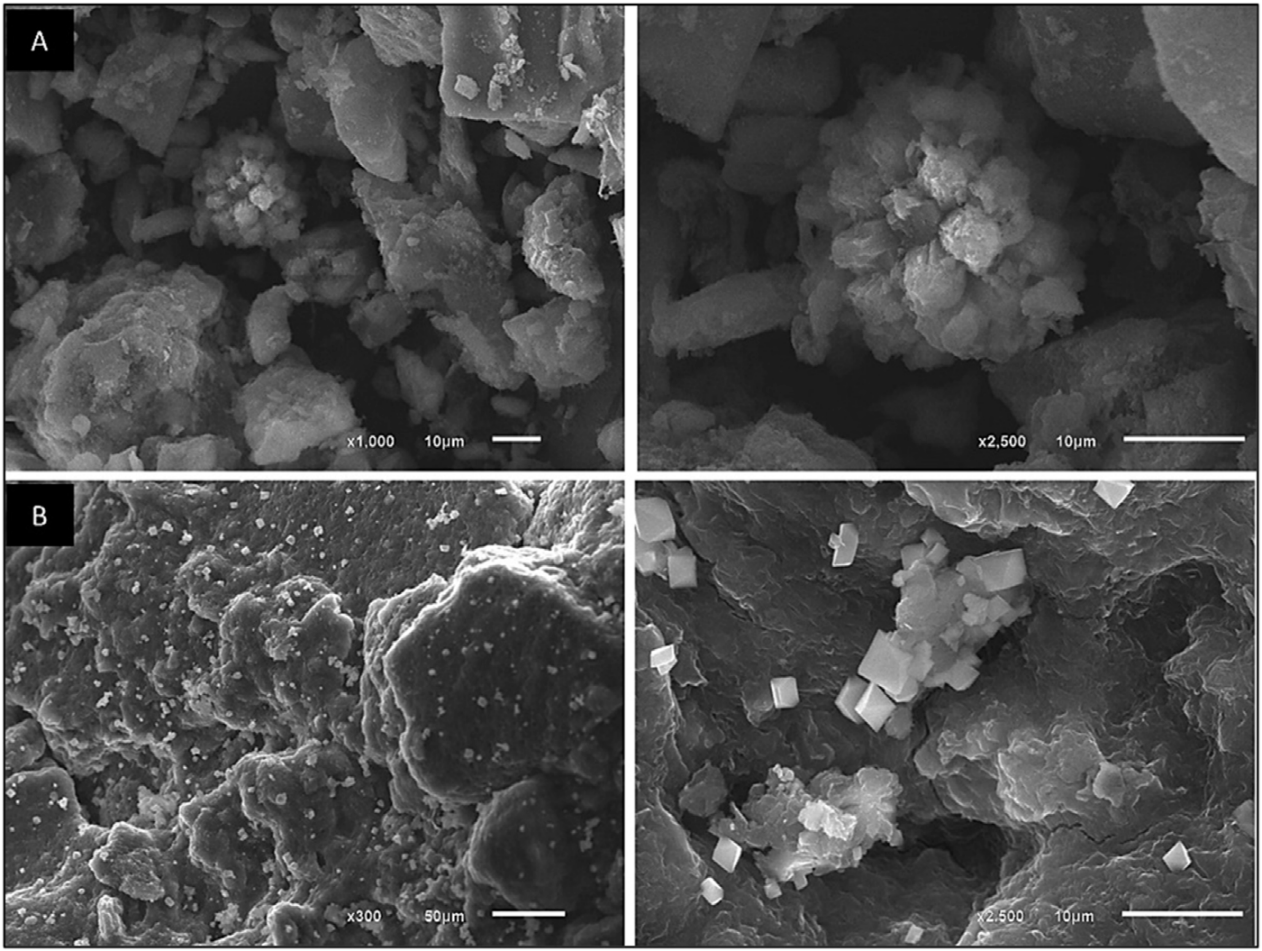
SEM images of **(A)** unloaded dried microalgae at 1,000× and 2,500× magnification, and **(B)** Cd^2+^-loaded biomass at 300× and 2500× magnification, illustrating morphological changes before and after cadmium adsorption.

SEM was utilized to investigate the morphological changes in dried microalgae before and after the adsorption process. As illustrated in Figure 13, the treated biomass cells demonstrated a noticeable increase in size compared to the untreated (blank) cells, indicating structural alterations likely induced by the adsorption of heavy metals. The surface of the treated biomass revealed the presence of numerous embedded solid crystals, characterized by a small plaque-like morphology, which were not observed on the untreated biomass.

The appearance of these crystalline deposits suggests a reduction mechanism during the biosorption process, whereby cadmium ions are potentially transformed and precipitated onto the biomass surface in a crystalline form. This transformation highlights the role of the biosorbent’s surface in capturing and retaining metal ions. The observed increase in cell size and the formation of crystal deposits further support the efficiency of the biosorption process and underline the capability of Chlamydomonas biomass as a promising material for heavy metal remediation.

#### 3.6.4 EDX analysis

**FIGURE 14:**
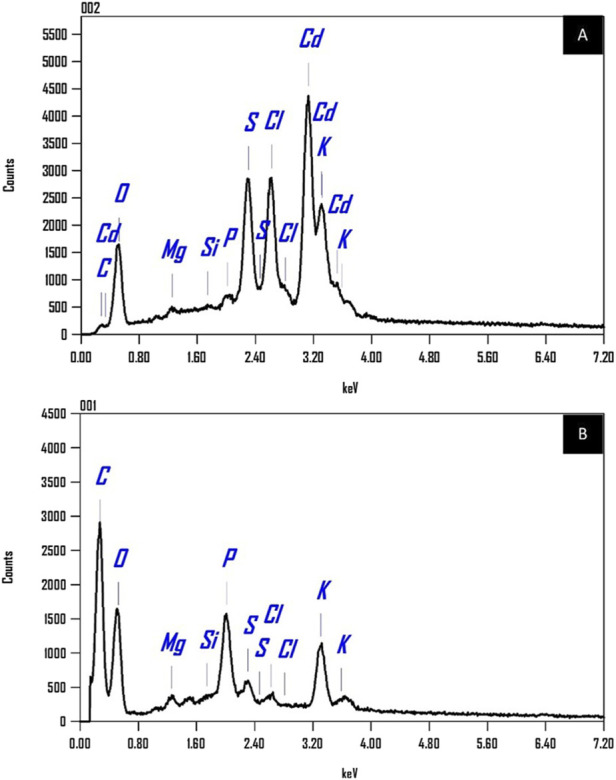
EDX spectra **(A)** for biomass with controlled cadmium loading, illustrating peaks indicative of cadmium presence, and **(B)** for biomass without cadmium loading, highlighting the absence of cadmium peaks.

Figure 14 displays the EDX spectra illustrating the elemental makeup of *Chlamydomonas* sp. biomass. The spectrum A, acquired post-cadmium biosorption, distinctly exhibits characteristic peaks for cadmium (Cd) at roughly 0.7, 3.1, and 3.7 keV, thereby affirming the successful attachment of cadmium ions to the biomass surface. Spectrum B, obtained after treatment to cadmium, exhibits no cadmium peaks. Both spectra indicate the existence of additional elements inherently present in the microalgal biomass, such as carbon (C), oxygen (O), magnesium (Mg), silicon (Si), phosphorus (P), sulfur (S), chlorine (Cl), and potassium (K). A comparison of peak intensities between the two spectra reveals significant changes indicative of elemental interactions during cadmium adsorption. A reduction in the intensity of the potassium (K) peak in Spectrum A, compared to Spectrum B, is noted. This decrease corroborates the notion of ion exchange, wherein K^+^ ions are substituted by Cd^2+^ ions during the biosorption process. The EDX data convincingly confirm the efficient absorption of cadmium by *Chlamydomonas* sp. biomass, offering substantial proof of its biosorption capability. They propose that various processes, including coordination and ion exchange, facilitate cadmium binding. The EDX mapping further validates this, indicating that cadmium is evenly distributed across the surface of the dry biomass, highlighting the efficacy of the biosorption process.

#### 3.6.5 Zeta potential and dynamic light scattering

Zeta potential values exhibited a considerable dependence on pH levels and positively influenced the sorption capability of the biosorbent. With an increase in pH, there was a corresponding rise in zeta potential values, demonstrating how the surface charge of the dried biomass varied with pH. The zeta potential values for the dried biomass at pH levels of 3, 4, 5, 6, 7, and 8 were noted as −22 mV, −34.6 mV, −35.5 mV, −39.2 mV, −39.4 mV, and −39.9 mV, respectively, as indicated in (Supporting data Figure 1). The recorded zeta potential values mirror the behavior and stability of the charges associated with the potential shear plane of material particles as they move within a liquid medium. The maximal possible negative charge active site of the biosorbent augments its adsorption capacity, aligning with the findings detailed in the pH optimization section. Consequently, the optimal pH for the biosorbent was determined to be 4, substantiated by the zeta potential data. The transition from a negative charge value of −22 mV at pH 3 to −34.6 mV at pH 4 implies that pH 4 is optimal for the adsorption of positively charged heavy metal ions. Additionally, as previously mentioned, the proposed adsorption mechanism, based on the apparatus utilized for the characterization of the biosorbent, aids in diminishing the ion exchange. The dynamic light scattering (DLS) technique was employed to assess the particle size uniformity and its influence on the heavy metal removal rate, which is a standard procedure in such evaluations. The homogeneity and monodispersity of biomass particles were verified utilizing the DLS technique. As demonstrated in the supporting data (Figure 2), the average dry biomass particle size was determined to be 342 nm.

### 3.7 Regeneration and reuse

**FIGURE 15:**
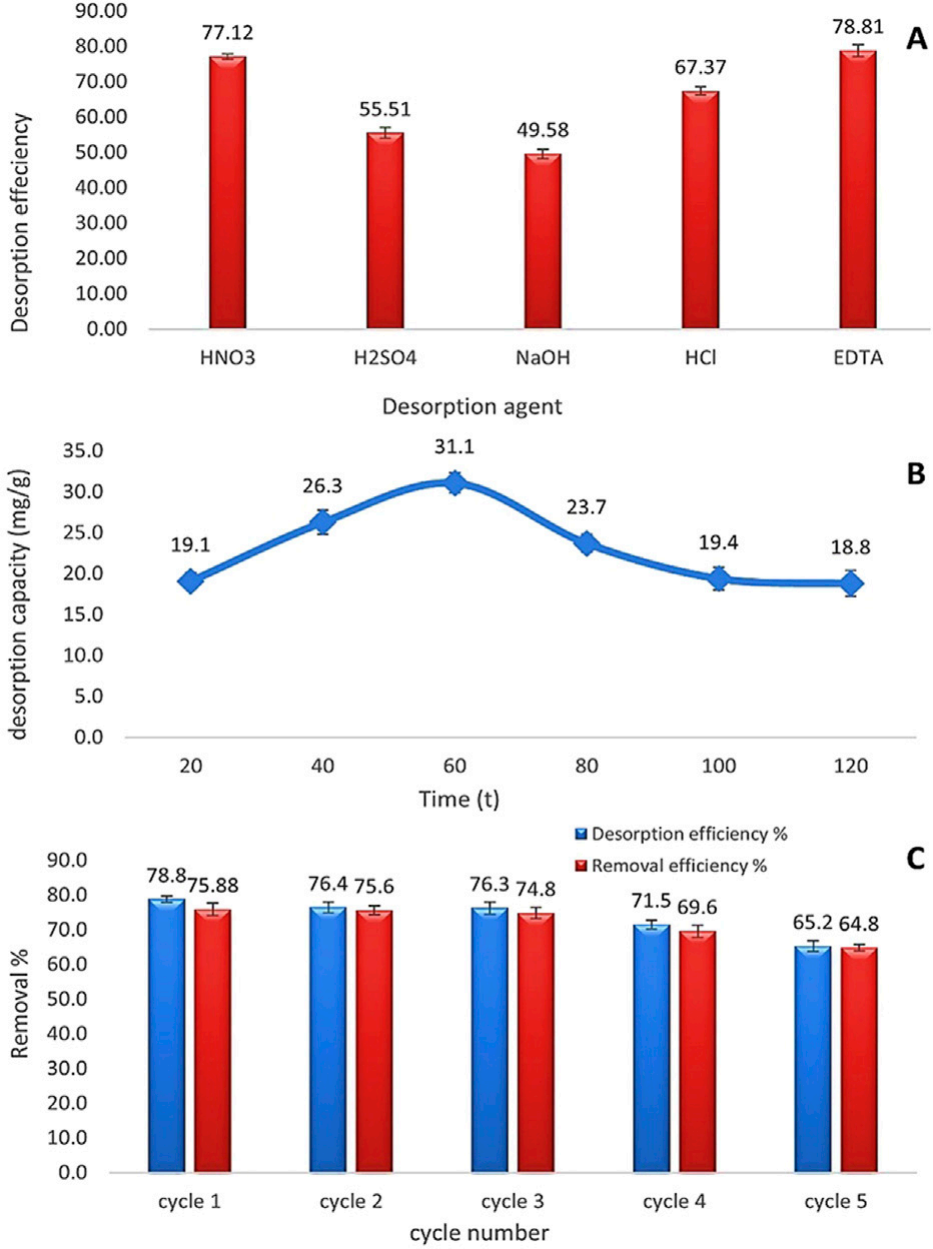
**(A)** Comparison of desorption efficiency (%) among different eluent agents (HNO_3_, H_2_SO_4_, NaOH, HCl, and EDTA). **(B)** Change in desorption capacity (mg/g) over time (min) using EDTA as the eluent agent. **(C)** Performance of EDTA as an eluent agent across five regeneration cycles. All experiments were conducted with a Cd2+ concentration of 25 mg/L, a biomass dose of 0.8 g/L, at a temperature of 25°C, pH 4, and a contact time of 60 min.

The preliminary phase entailed assessing the selectivity of the optimal eluent agent following the determination of the feasibility of reutilizing dried microalgae as a biosorbent. Desorption efficiency was evaluated through experimentation with various eluent agents, including HNO_3_, H_2_SO_4_, HCl, 0.1 NaOH, and EDTA, as illustrated in Figure 15. The results illustrated that acidic eluent agents (HNO_3_: 77.12%, H_2_SO_4_: 55.51%, and HCl: 67.37%) were the most efficacious, attributed to the presence of protons. Conversely, 0.1 NaOH proved to be the least effective, exhibiting a desorption efficiency of 49.58%. EDTA emerged as the most effective desorbing agent, with a desorption efficiency of 78.81% owing to its chelating properties. An additional experiment was performed to ascertain the optimal contact time for the desorption process, which was determined to be 60 min. Subsequently, five cycles of the same eluent agent were conducted, revealing that the adsorption efficacy of *Chlamydomonas* as a dried biosorbent was 64.8% after the fifth cycle, with a consistent desorption efficacy of 65.2% throughout each cycle.

## 4 Discussion

### 4.1 Mechanism of cadmium adsorption by microalgae

The present study’s findings suggest that the removal of cadmium using dried *Chlamydomonas* biomass is notably efficient under optimized conditions. At an optimal pH of 4 and a biosorbent concentration of 0.8 g/L, the biosorption process achieved a maximum removal efficiency of 81.5%. Correspondingly, temperature assessments indicated that an 81.5% removal rate of cadmium was attained at the optimal temperature of 25°C, with a slight decline observed at temperatures deviating from this optimal point. The efficiency of removal was significantly influenced by the initial concentration of cadmium, exhibiting a high removal efficiency of 95.6% at an initial concentration of 25 mg/L, which diminished to 67.2% at 50 mg/L and further decreased at elevated concentrations. These findings suggest interactions between cadmium ions and functional groups such as (OH), (COOH), and (NH_2_) present on the surface of the biosorbent (Komy et al., 2006). FTIR analysis demonstrated the participation of these groups through notable shifts and broadening of peaks following adsorption, indicative of mechanisms such as ion exchange, electrostatic attraction, and coordination bonding (Gonzalez, 2001). XRD analysis further revealed structural alterations within the biomass, encompassed peak changes, and the emergence of new peaks, which imply the formation of cadmium complexes and structural rearrangements. SEM results further revealed augmented surface roughness and crystalline depositions on the biomass, corroborating the occurrence of cadmium precipitation or complexation.

Microalgae, such as *Chlamydomonas reinhardtii*, have demonstrated efficacy in the removal of cadmium from aqueous solutions. It has been documented that fresh cells are capable of absorbing up to 42.6 mg/L of cadmium, whereas lyophilized biomass can achieve a removal rate of up to 67.4 mg/L (Mohamed et al., 2021). This substantial uptake is attributed to the distinctive cellular structures of the microalgae, which permit the binding and sequestration of metal ions. The biosorption process predominantly involves passive adsorption mechanisms, wherein cadmium ions engage with functional groups present on the algal cell surface, including carboxyl, hydroxyl, and amino groups (Xu et al., 2022). The intracellular mechanisms underlying cadmium accumulation in fresh microalgae cells encompass intricate transport systems and biochemical pathways. Cadmium ions may penetrate algal cells via specific transporters that are likewise implicated in the acquisition of essential nutrients. This occurrence frequently results in competitive inhibition, wherein cadmium disrupts the uptake of vital elements such as calcium and zinc, thereby inducing physiological stress (Falco et al., 2010). Furthermore, the sequestration of cadmium within vacuoles and the synthesis of metal-binding proteins, such as phytochelatins, are pivotal in detoxifying cadmium and averting its detrimental effects on cellular functions (Penen et al., 2019). Furthermore, the interaction of cadmium with algal cells has the potential to induce morphological and physiological alterations. For example, exposure to cadmium has been documented to disrupt the cytoskeleton of microalgae, thereby impacting their growth and metabolic functions (Hussain et al., 2020). Additionally, the production of extracellular polymeric substances by microalgae can augment cadmium biosorption by providing supplementary binding sites for metal ions, which ultimately enhances the efficiency of the bioremediation process (Boonchai et al., 2015).

In summary, cadmium adsorption onto *Chlamydomonas* sp. transpires via two primary mechanisms, with chemisorption as the predominant route. Physical adsorption, characterized by pore diffusion, as demonstrated by SEM examination revealing porous biomass structures, and weak van der Waals contacts (Nouacer et al., 2023). The principal mechanism is chemical binding, wherein cadmium ions specifically couple with functional groups on the algal surface, particularly carboxyl (–COOH) and hydroxyl (–OH) groups, as indicated by notable FTIR spectrum shifts at 1,650 cm^−1^ and 3,400 cm^−1^, respectively (Sen, 2023). The dominance of chemisorption is additionally corroborated by kinetic data that conform to a pseudo-second-order model. The observed ion exchange processes, wherein cadmium displaces lighter metal ions (Na^+^, K^+^) as evidenced by EDX analysis, illustrates the irreversible nature of the binding process. Moreover, polymeric compounds in microalgae aid in cadmium sequestration by offering supplementary binding sites and promoting the development of stable metal-ligand complexes, hence augmenting the biosorption efficacy of *Chlamydomonas* sp. for cadmium elimination (Alyasi et al., 2019). Moreover, research indicates that altering dried microalgal biomass by methods like acid treatment or the addition of nanoparticles enhances the accessibility of reactive sites, thus augmenting its adsorption capability (Peng et al., 2018).

### 4.2 Optimization of biosorption parameters

The biosorption capacity of microalgae is influenced by various factors, including pH, temperature, and the concentration of cadmium in the surrounding environment. For instance, a previous study conducted on *Tetraselmis suecica*, exhibited high tolerance to cadmium and has been extensively studied for its biosorption properties. The sorption isotherm studies conducted on this species reveal that cadmium ions can effectively bind to the algal biomass, indicating a potential for water purification applications (Pérez-Rama et al., 2010). Furthermore, the physiological responses of microalgae to cadmium exposure, such as the production of reactive oxygen species and the activation of antioxidant mechanisms, play a crucial role in mitigating metal toxicity and enhancing biosorption efficiency (Hussain et al., 2020).

The ionization of functional groups present on the microalgal cell wall, particularly carboxyl and hydroxyl groups, is dependent on pH levels. Previous research indicated that optimal pH levels for heavy metals biosorption are generally within the range of 4–7, with acidic conditions frequently enhancing the uptake of metals due to the increased availability of binding sites (Ayele et al., 2021). For instance, *Chlamydomonas* sp. achieved a maximum biosorption efficiency of 94.17% for chromium (VI) at a pH of 4, which is markedly higher than that observed at alkaline pH levels (Ayele et al., 2021). In a similar vein, *Spirogyra porticalis* demonstrated enhanced efficiencies in the removal of chromium ions at lower pH levels, suggesting that acidic conditions can improve the accessibility of metal ions in aqueous environments (Sayyaf et al., 2015).

Additionally, biomass dose constitutes a pivotal parameter affecting the biosorption capacity of microalgae. An increase in biomass concentration generally augments the removal efficiency of heavy metals, attributable to the enhanced availability of binding sites. Nevertheless, there exists a threshold where further augmentation in biomass may result in diminished efficiency, attributed to particle aggregation and restricted access of metal ions to the binding sites (Saurav and Kannabiran, 2011). For example, optimal biosorption of cadmium was demonstrated at a biomass dose of 0.5 g/L, beyond which the efficiency reached a plateau (Saurav and Kannabiran, 2011). This phenomenon underscores the significance of determining the optimal biomass concentration specific to each microalgal species and the particular heavy metal being targeted.

Temperature exerts a critical influence on the biosorption process by impacting the metabolic activity of microalgae and the kinetics of metal ion adsorption. Elevated temperatures can facilitate an increased diffusion rate of metal ions toward the biomass, thereby augmenting the overall biosorption capacity. Nonetheless, excessively high temperatures may also prompt the denaturation of proteins and other biomolecules implicated in the adsorption process (Sultana et al., 2020). Empirical studies have demonstrated that optimal temperatures for cadmium biosorption generally lie within the range of 25°C–35°C, where the metabolic processes of microalgae reach peak efficiency (Xu et al., 2022). For example, Dunaliella salina achieved effective lead removal at approximately 30°C, indicating that moderate thermal conditions are favorable for optimal biosorption (Ziaei et al., 2023).

The initial concentration of heavy metals in the solution is a critical determinant affecting the biosorption capacity of microalgae. Elevated initial concentrations generate a more substantial driving force for mass transfer, thereby potentially increasing the rate of metal uptake. However, this relationship is not linear, as saturation of the binding sites may occur at higher concentrations, leading to reduced removal efficiencies (El-Gendy et al., 2023). For example, the biosorption of cadmium by Scenedesmus sp. showed significant increases in removal efficiency with initial concentrations up to 100 mg/L, beyond which the efficiency began to decline (Monteiro et al., 2011). This highlights the necessity of optimizing initial metal concentrations to achieve maximum biosorption while avoiding saturation effects.

### 4.3 Kinetics, equilibrium, and thermodynamics of Cd^2+^ adsorption

The process of cadmium bioadsorption by microalgae has produced substantial insights into the kinetics, equilibrium, and thermodynamics involved, thus providing a thorough comprehension of the fundamental mechanisms. The kinetics of cadmium uptake in microalgae are affected by various factors, such as the external cadmium concentration and the physiological conditions of the cells. For example, *Scenedesmus obliquus* demonstrates a dynamic response to cadmium exposure, wherein interactions between algal organic matter and cadmium ions trigger detoxification mechanisms that enhance metal uptake (Xu et al., 2024). The kinetics of cadmium adsorption can be accurately characterized using models such as the Langmuir and Freundlich isotherms. Nevertheless, the Elovich and intraparticle diffusion models provide essential insights into the adsorption process, highlighting an initial rapid adsorption phase succeeded by slower pore diffusion and ultimately reaching equilibrium. These observations underscore diffusion as a secondary rate-limiting step, consistent with kinetic studies on analogous biosorbents. The Langmuir isotherm model has been recognized as the most appropriate for characterizing cadmium adsorption on microalgal biomass, suggesting monolayer adsorption on a uniform surface. The maximum adsorption capacity of 42.9 mg/g highlights the efficiency of the biosorbent and its potential for practical applications. Conversely, the Freundlich, Temkin, and Dubinin-Radushkevich models exhibit a less satisfactory fit, potentially due to the uniformity of the biosorbent surface. These observations reinforce the significance of the Langmuir model in elucidating the adsorption characteristics of microalgae. Thermodynamic analyses reveal that the cadmium adsorption process is spontaneous, as evidenced by the negative Gibbs free energy values. The positive enthalpy corroborates the endothermic nature of the adsorption mechanism, while the positive entropy indicates increased randomness at the solid-liquid interface during the process. These parameters underscore the thermal dependency of biosorption and emphasize the importance of optimizing temperature conditions for industrial applications.

### 4.4 Influence of competing ions

Monovalent ions such as Na^+^ and K^+^ are generally observed to exert an insignificant influence on cadmium biosorption. This phenomenon is primarily attributable to their reduced charge density compared to divalent ions, which leads to diminished electrostatic interactions with the negatively charged sites on the microalgal cell walls. Empirical evidence has demonstrated that the presence of Na^+^ and K^+^ does not substantially impede cadmium uptake, as these ions do not effectively compete for the same binding sites (Umeh, 2023). The biosorption capacity is observed to remain relatively constant in the presence of these monovalent ions, signifying that they do not displace cadmium ions from the active sites on the microalgal biomass.

Conversely, divalent ions such as Ca^2+^ can exert a significant influence on cadmium biosorption due to their enhanced affinity for active sites on microalgae. The presence of Ca^2+^ facilitates competitive binding, in which cadmium ions are displaced from their binding sites, thus diminishing the overall biosorption capacity for cadmium. This occurrence is attributed to the elevated charge density of divalent ions, which enables them to establish stronger electrostatic interactions with the negatively charged functional groups on the algal surface (Mazur et al., 2018). Consequently, the presence of Ca^2+^ can markedly decrease the efficiency of cadmium removal from contaminated water. Additionally, the effect of competing ions on cadmium biosorption can be elucidated through the concept of electric double-layer (EDL) compression. An increase in ionic strength, attributed to the presence of competing ions, results in the compression of the EDL surrounding the microalgal biomass. This compression diminishes the effective range of electrostatic interactions, thereby influencing the accessibility of cadmium ions to binding sites (Zhang, 2024). The compression of the EDL may result in a reduction of the repulsive forces between the negatively charged algal surface and cadmium ions, ostensibly appearing advantageous. Nevertheless, the competitive binding of divalent ions such as Ca^2+^ may negate these advantages, culminating in reduced cadmium uptake.

### 4.5 Comparative analysis of dried *Chlamydomonas* and other adsorbents for Cd^2+^

Data presented in Table 2 compares dried *Chlamydomonas* sp. microalgae with several adsorbents that have been studied in the literature in terms of maximum adsorption efficiency (mg/g) and cadmium (II) removal percentage. The table highlights the significant variation in adsorbent values observed for different adsorbents. The results indicate that dried *Chlamydomonas* sp. exhibits favorable adsorption efficiency for cadmium ions from water-based solutions, outperforming different absorbents that have been examined in the literature, making it a promising candidate for further investigation.

**TABLE 2:** Comparison between *Chlamydomonas* as biosorbent in this study and other biosorbents in previous studies for cadmium (II).

Adsorbent	Conditions	Adsorption capacity (mg/g)	Removal efficiency %	Reference
Orange peel (OP)-derived biochar	Contact time: 129 min Dose: 6 gm/L Concentration: 100 mg/L pH: 9.5 Temperature: 25°C	114.69	96%	Tran et al. (2016)
Coated Chicken Bones with Double-Layer Hydroxide (Mg/Fe-LDH)	Contact time: 180 min Dose: 10 gm/100 mL Concentration: 20 mg/L pH: 5.0 Temperature: 25°C	-	97%	Alquzweeni and Alkizwini (2020)
*Cladophora* sp	Contact time: 60 min Dose: 0.2 gm/L Concentration: 50 mg/L pH: 4.0 Temperature: 32°C	12.07	80%	Amro and Abhary (2019)
Natural phosphate	Contact time: 120 min Dose: 4 gm/L Concentration: 50 mg/L pH: 5.0 Temperature: 25°C	26	-	Yaacoubi et al. (2014)
green synthesis iron oxide nanoparticles with tangerine peel extract	Contact time: 90 min Dose:0. 4 gm/100 mL Concentration: 5 mg/L pH: 4.0 Temperature: 25°C	-	90%	Ehrampoush et al. (2015)
unmodified and NTA-modified Dendrocalamus strictus charcoal powder	Contact time: 120 min Concentration: 1 mg/L pH: 6 Temperature: 25°C	166.66	91.47%	Saini et al. (2019)
*Chlamydomonas* sp	Contact time: 60 min Dose: 0.8 gm/L Concentration: 25–200 mg/L pH: 4.0 Temperature: 25°C	44.75	95.6%	This study

## 5 Conclusion

This study explored the potential of dried *Chlamydomonas* as an efficient biosorbent for cadmium removal from industrial wastewater. Through an optimization process, the key parameters influencing adsorption were identified, revealing optimal conditions of 25°C, pH 4, a cadmium concentration of 50 mg/L, a contact time of 60 min, and a biomass dose of 0.8 g/L. Under these conditions, the biosorption capacity reached an impressive 44.75 mg/g, with a maximum cadmium removal efficiency of 95.6%. These results highlight the viability of dried *Chlamydomonas* as a cost-effective and sustainable solution for addressing heavy metal contamination in water systems. The findings emphasize the importance of optimizing biosorption parameters such as pH, biomass dosage, temperature, and metal concentration to enhance removal efficiency. Each parameter uniquely influences the biosorption process, and their interactions often present challenges for optimization. This promising approach offers a significant step toward mitigating heavy metal pollution in wastewater treatment systems.

## Data Availability

The original contributions presented in the study are included in the article/Supplementary Material, further inquiries can be directed to the corresponding authors.
