# Supplementary material for: Phycomediation of cadmium contaminated aqueous solutions using Chlamydomonas sp.: process optimization and adsorption characterization
Source: Front Bioeng Biotechnol. 2025 Mar 26;13:1558757. doi: 10.3389/fbioe.2025.1558757 (PMC11979156; doi:10.3389/fbioe.2025.1558757)
Supplement: Supplementary file 1 [file DataSheet1.PDF]

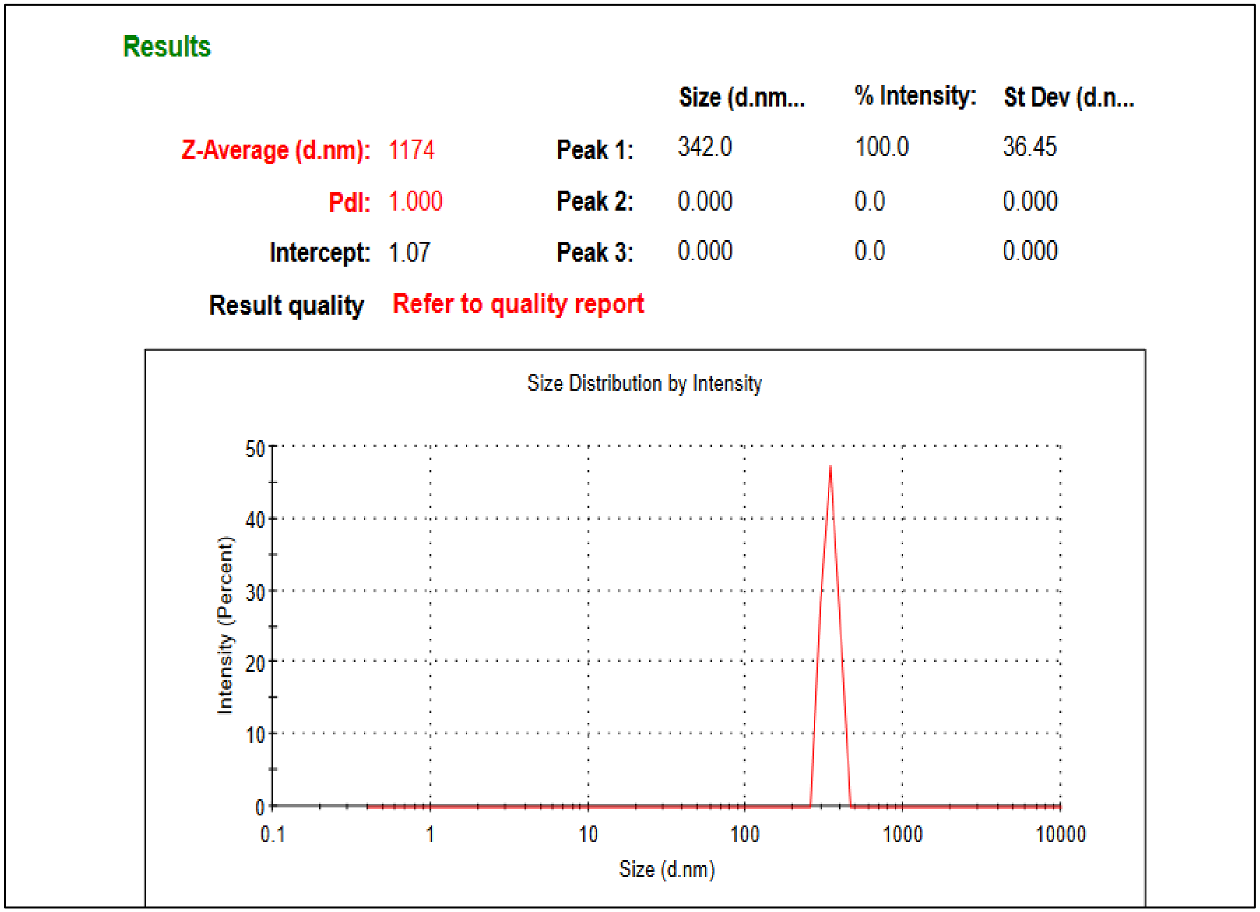

Fig. 1. DLS chart for dried Chlamydomonas sp. biomass showing average particle size 1174 nm and PDI = 1.

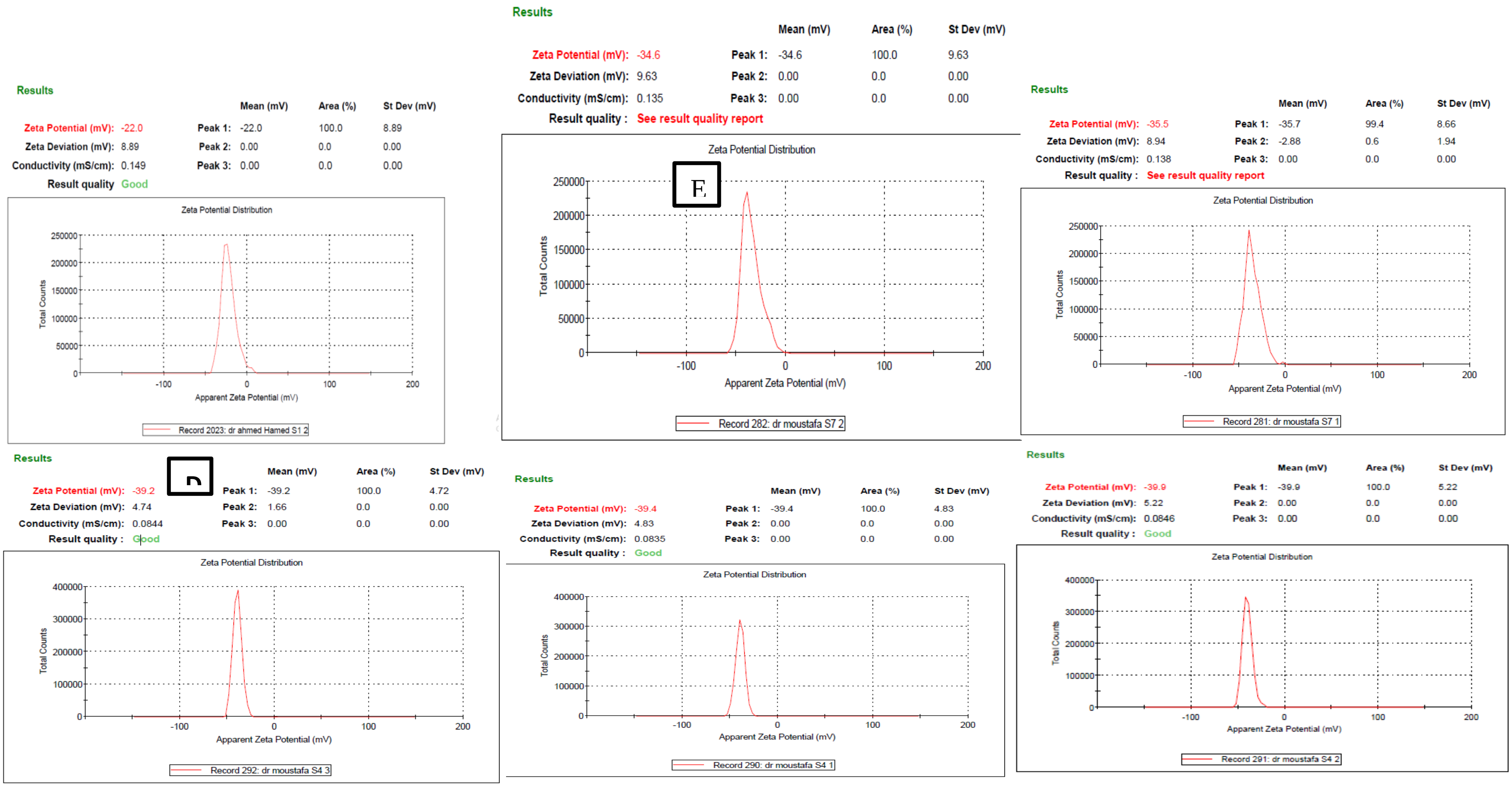

Fig. 2. Zeta potential chart for (A) dried Chlamydomonas sp. biomass at pH 3, recorded -22 mV potential and 0.149 mS/cm conductivity. (B) for dried Chlamydomonas sp. biomass at pH 4, recorded -34.6 mV potential and 0.135 mS/cm conductivity. (C) for dried Chlamydomonas sp. biomass at pH 5, recorded -35.5 mV potential and 0.138 mS/cm conductivity. (D) for dried Chlamydomonas sp. biomass at pH 6, recorded -39.2 mV potential and 0.0834 mS/cm conductivity. (E) for dried Chlamydomonas sp. biomass at pH 7, recorded -39.4 mV potential and 0.0835 mS/cm conductivity. (F) for dried Chlamydomonas sp. biomass at pH 8, recorded -39.9 mV potential and 0.0846 mS/cm conductivity.

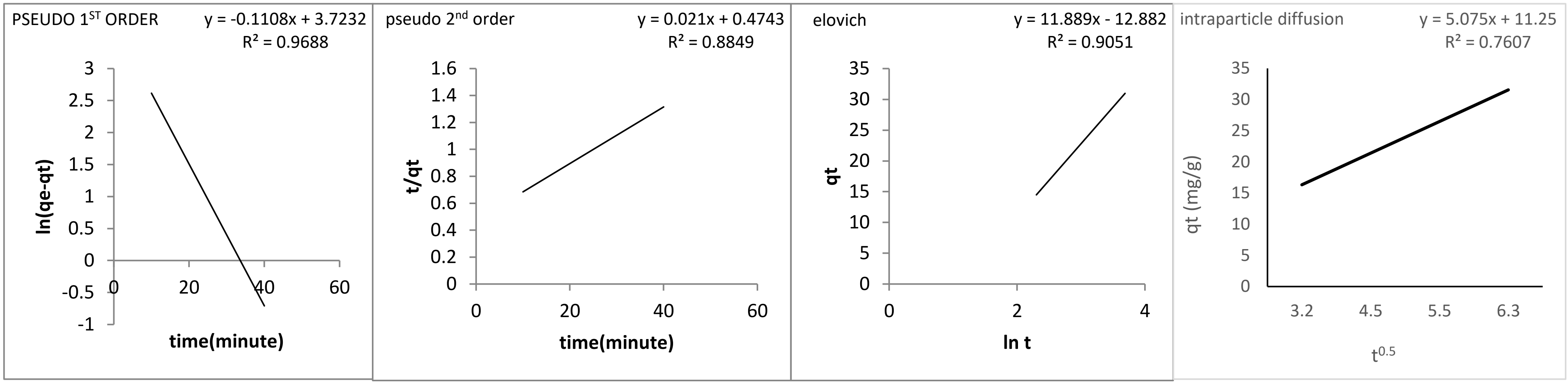

Fig. 3. Kinetic models 1ST, 2nd order, Elovich and intraparticle diffusion for initial cadmium (II) concentration 25 mgl^{-1}.

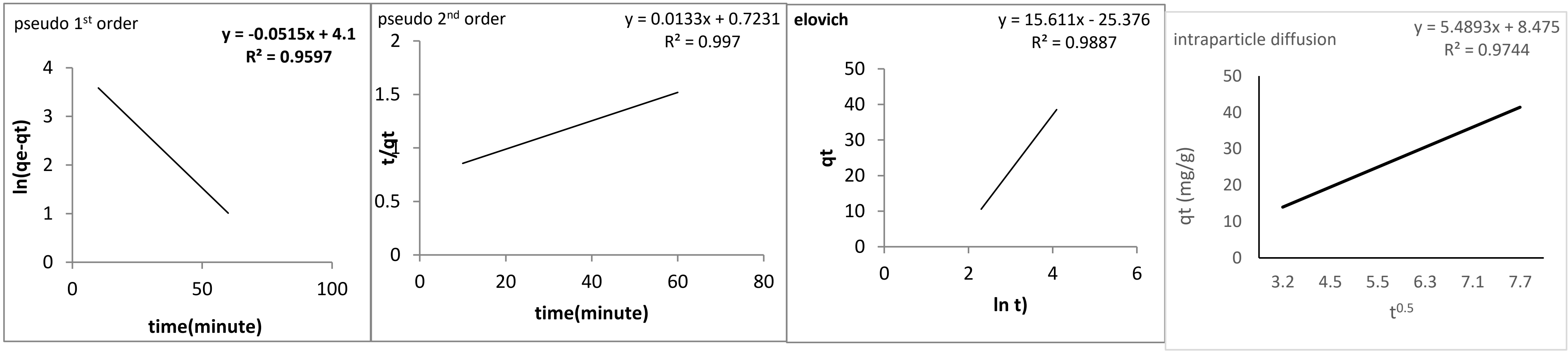

Fig. 4. kinetic model's 1ST, 2nd order, Elovich and intraparticle diffusion for initial cadmium (II) concentration 50 mgl^{-1}.

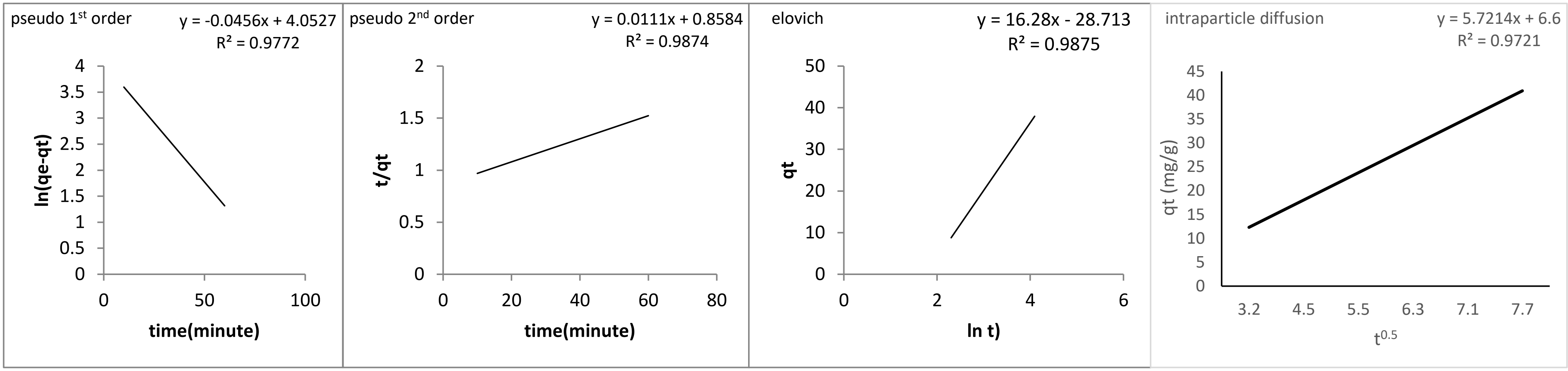

Fig. 5. kinetic model's 1ST, 2nd order, Elovich and intraparticle diffusion for initial cadmium (II) concentration 100 mgl^{-1}.

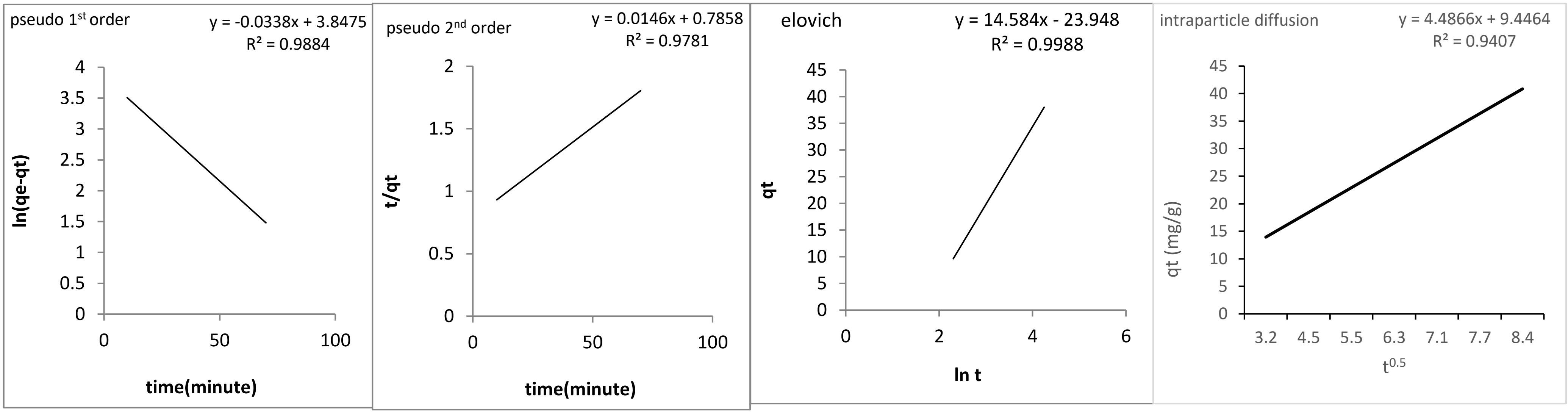

Fig. 6. Kinetic models 1ST, 2nd order, Elovich and intraparticle diffusion for initial cadmium (II) concentration 140 mgl^{-1}.

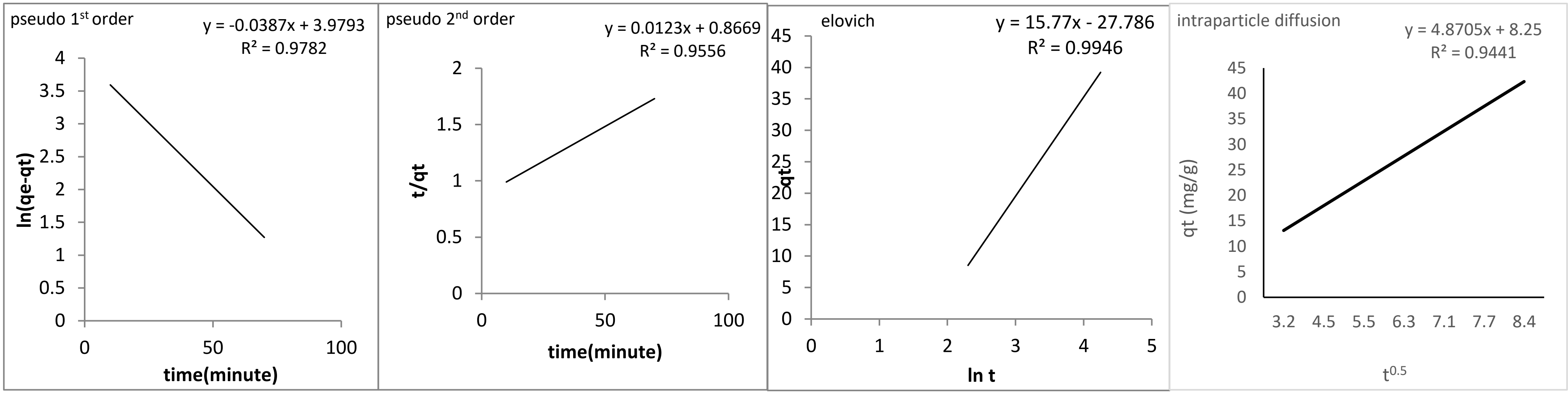

Fig. 7. Kinetic models 1ST, 2nd order, Elovich and intraparticle diffusion for initial cadmium (II) concentration 180 mgl-1.

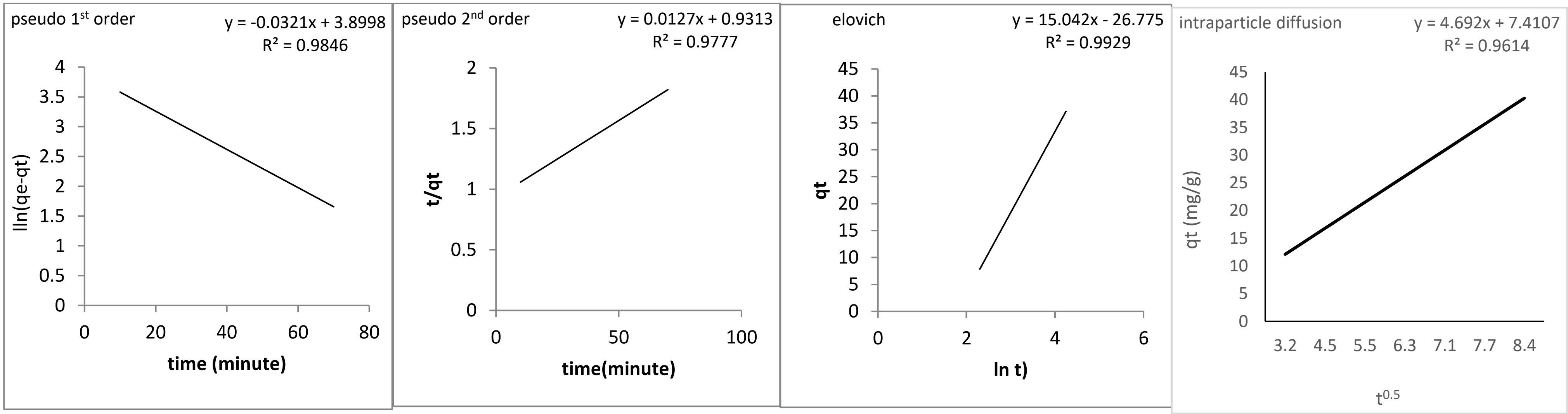

Fig. 8. Kinetic models 1ST, 2nd order, Elovich and intraparticle diffusion for initial cadmium (II) concentration 200 mgl-1.

Table 1. Effect of contact time on biosorption capacity at variable cadmium (II) concentration, constant pH 4, and constant dried biomass 0.8 gm/l.

t (minute)	Removal percentage (%) for 25 mg/l	q_t for 25 mg/l	±SD	Removal percentage (%) for 50 mg/l	q_t for 50 mg/l	±SD	Removal percentage (%) for 100 mg/l	q_t for 100 mg/l	±SD	Removal percentage (%) for 140 mg/l	q_t for 140 mg/l	±SD	Removal percentage (%) for 180 mg/l	q_t for 180 mg/l	±SD	Removal percentage (%) for 200 mg/l	q_t for 200 mg/l	±SD
10	41.2	12.9	1.4	18.8	11.8	1.4	8.1	10.1	1.4	5.6	9.9	2.6	4.1	9.3	1.4	3.6	8.9	1.4
20	82	25.6	1.8	32.6	20.4	1.8	14.8	18.5	1.8	11.3	19.8	1.4	8.4	19.0	1.8	7.1	17.8	1.4
30	90.8	28.4	1.63	42.2	26.4	1.63	20.6	25.8	1.63	14.5	25.4	1.4	10.8	24.4	1.63	9.2	23.0	1.3
40	92.4	28.9	2.4	50.6	31.6	2.4	24.5	30.6	2.4	16.9	29.6	1.3	13.8	31.0	2.4	11.4	28.5	0.9
50	94.4	29.5	2.6	58.6	36.6	2.6	28.9	36.1	2.6	18.6	32.6	0.9	15.2	34.3	2.6	12.8	32.0	1.1
60	94.8	29.6	1.4	63	39.4	1.4	30.9	38.6	1.4	20.6	36.0	1.1	16.2	36.5	1.4	14.0	34.9	1.6
70	95.2	29.8	1.4	66.4	41.5	1.4	33.5	41.9	1.4	22.0	38.5	1.6	17.7	39.8	1.63	15.3	38.3	1.3
80	95.2	29.8	1.3	66.8	41.8	1.3	33.5	41.9	1.63	24.2	42.4	1.3	18.9	42.6	2.4	17.1	42.8	1.4
90	95.2	29.8	2.4	67	41.9	0.9	33.5	41.9	2.4	24.2	42.4	0.9	19.1	43.0	2.6	17.4	43.5	1.8
100	95.2	29.8	2.6	67	41.9	1.1	33.9	42.4	2.6	24.2	42.4	1.1	19.1	43.0	1.4	17.4	43.5	1.63
110	95.2	29.8	1.4	67	41.9	1.6	33.9	42.4	1.4	24.5	42.9	1.6	19.1	43.0	1.4	17.6	43.9	2.4
120	95.2	29.8	1.4	67	41.9	1.1	33.9	42.4	1.4	24.5	42.9	1.1	19.2	43.3	2.3	17.6	43.9	1.1
130	95.2	29.8	2.3	67	41.9	2.3	33.9	42.4	2.3	24.5	42.9	2.3	19.2	43.3	1.9	17.6	43.9	2.3
140	95.6	29.9	1.9	67	41.9	1.9	33.9	42.4	1.9	24.9	43.6	1.9	19.5	43.9	1.4	17.8	44.4	1.9
150	95.6	29.9	1.4	67	41.9	1.4	34.1	42.6	1.4	24.9	43.6	1.4	19.5	43.9	1.4	17.8	44.5	1.4
160	95.6	29.9	1.4	67.2	42.0	1.6	34.1	42.6	1.6	24.9	43.6	1.6	19.5	43.9	1.6	17.9	44.8	1.6

*Where t is time (minute), q_t is biosorption capacity at a certain time (mg/g), and SD is standard deviation.

Table 2. Values of 1ST order kinetic models.

initial conc. (mg/l)	intercept	slope	q max measured (mg/g)	q max (mg/g)	R^2	k_1
**25**	3.27	-0.1108	39.67	26.40	0.9688	-0.033850666
**50**	4.10	-0.0515	51.83	60.34	0.9597	-0.012560976
**100**	4.95	-0.0456	51.17	141.56	0.9772	-0.009207099
**140**	3.85	-0.0338	51.83	46.88	0.9884	-0.008784925
**180**	3.94	-0.0321	50.67	51.57	0.9457	-0.008141009
**200**	3.90	-0.0321	50.17	49.39	0.9846	-0.008231191

*Where q_{max} is biosorption capacity (mg/g). K_1 is the pseudo-first-order rate constant for the kinetic model (1/min) and R^2 is the regression correlation coefficient.

Table 3. Values of 2nd-order kinetic models.

initial conc. (mgl^{-1})	intercept	slope	q max measured (mg/g)	q max (mg/g)	q max ^{2}	k_2	R^2
**25**	0.4743	0.0214	39.67	46.73	2183.596821	0.000965549	0.9688
**50**	0.7231	0.0133	51.83	75.19	5653.230821	0.000244627	0.997
**100**	0.8584	0.0111	51.17	90.09	8116.224332	0.000143534	0.9874
**140**	0.7858	0.0146	51.83	68.49	4691.311691	0.000271265	0.9781
**180**	0.9041	0.0144	50.67	69.44	4822.530864	0.000229355	0.9435
**200**	0.9313	0.0127	50.17	78.74	6200.0124	0.000173188	0.9777

*Where qmax is biosorption capacity (mg/g), K_2 is the 2nd order rate constant for the kinetic model (g $mg^{-1}min^{-1}$) and R^2 is the regression correlation coefficient.

Table 4. Values of elovich kinetic models.

Initial conc. (mg/l)	slope	inter	β (g /mg^{-1})	α (mg g^{-1} min^{-1})	R2
**25**	11.889	-12.882	0.084111363	4.023253964	0.9051
**50**	15.611	-25.376	0.064057395	3.072388671	0.9887
**100**	16.28	-28.713	0.061425061	2.790552408	0.9875
**140**	14.584	-23.948	0.068568294	2.823145058	0.9988
**180**	14.182	-24.183	0.070511917	2.577407059	0.9733
**200**	15.042	-26.775	0.066480521	2.536614538	0.9929

*Where β is desorption constant (g /mg^{-1}), α is the initial adsorption rate (mg g^{-1} min^{-1}) and R^2 is the regression correlation coefficient.

Table 5. Values of intraparticle diffusion kinetic models.

initial conc (mg/l)	**k_{diff} (mg g^{-1}·min$^{-0.5}$)**	**intercept**	**R^2**
**25**	5.075	11.25	0.7607
**50**	5.4893	8.475	0.9744
**100**	4.4866	6.6	0.9721
**140**	4.8075	9.4464	0.9407
**180**	4.8705	8.25	0.9441
**200**	4.692	7.4107	0.9614

*Where K_{diff} is the intraparticle diffusion constant (mg g^{-1}min$^{-0.5}$), R^2 is the initial adsorption rate (mg/g. min) and R^2 is the regression correlation coefficient.

Table 6. Calculated values for plotting Langmuir, Freundlich, Temkin, and Dubinin-Radushkevich isotherm models.

Exp. no.	Ci (mg/l)	Ce (mg/l)	qe (mg/g)	1/Ce	ln Ce	Ce/qe	log Ce	1/qe	log qe	log Ce	R(J/mol k)	Ln qe	T+273.15	1+(1/Ce)	ln(1+(1/ce))	RTln1+(1/Ce)	$Ɛ^2$
**1**	25	1.4	29.5	0.714	0.336	0.047	0.146	0.034	1.470	0.146	8.314	3.384	262.150	1.714	0.539	1174.751	1380039.941
**2**	50	16.8	41.5	0.060	2.821	0.405	1.225	0.024	1.618	1.225	8.314	3.726	262.150	1.060	0.058	126.019	15880.695
**3**	100	66.5	41.875	0.015	4.197	1.588	1.823	0.024	1.622	1.823	8.314	3.735	262.150	1.015	0.015	32.531	1058.245
**4**	140	106.1	42.375	0.009	4.664	2.504	2.026	0.024	1.627	2.026	8.314	3.747	262.150	1.009	0.009	20.446	418.034
**5**	180	145.9	42.625	0.007	4.983	3.423	2.164	0.023	1.630	2.164	8.314	3.752	262.150	1.007	0.007	14.887	221.636
**6**	200	165.8	42.75	0.006	5.111	3.878	2.220	0.023	1.631	2.220	8.314	3.755	262.150	1.006	0.006	13.106	171.766

*Where Ci is the initial heavy metal concentration (mg/l), Ce is the heavy metal concentration at equilibrium (mg/l), R is the universal gas constant (g/mol k), T is the temperature in kelvin k, $Ɛ^2$ is the square of potential energy and qe is biosorption capacity at equilibrium (mg/g).

Table 7. R2 and constants for Langmuir, Freundlich, Temkin and Dubinin-Radushkevich isotherm models.

**Langmuir**			**Freundlich**			**Temkin**			**Dubinin – Radushkevich**			
q_{max} (mg/g)	K_L (l/mg)	R^2	k_f (mg/g)	1/n	R^2	k_t (l/mg)	B (J/mol)	R^2	q_{max} (mg/g)	E (KJ/mol)	k_{DR} (mol^2/kj^2)	R^2
42.91	1.14	0.9998	30.32	0.0737	0.8513	1.96	2.0036	0.5164	48.32	2.9988	-0.0556	0.5025

*Where q_{max} is the maximum adsorption capacity (mg/g), K_L is Langmuir constant (l/mg), K_f is freundich constant (mg/g), kt is temkin constant (l/mg), k_{DR} is Dubinin–Radushkevich isotherm constant(mol^2/kj^2) , 1/n is the empirical constant, R^2 is the regression correlation coefficient and E is energy of adsorption.

Table. 8. Values of thermodynamic study.

t °C	T (K)	K (K^{-1})	ΔG∘ (KJ mol^{-1})	ΔH∘ (KJ mol^{-1})	ΔS∘ ($JK^{-1}mol^{-1}$)	R^2
15	288	1.021303258	0.178285768	17.5857728	63.0417364	0.5265
20	293	1.079470199	-0.18628196			
25	298	2.341954023	-2.108378178			
30	303	2.047738693	-1.805560032			
35	308	1.968599034	-1.73442693			
40	313	1.949760766	-1.737561077			

* Where T is the temperature (K) and K is the distribution coefficient, (ΔG°) is energy (KJ mol^{-1}), (ΔH°) the enthalpy (KJ mol^{-1}), (ΔS°) entropy ($JK^{-1}mol^{-1}$) and R^2 is the regression correlation coefficient.

Table 9. XRD Peaks data features of controlled Cd (II) unloaded biomass and controlled Cd (II) loaded biomass.

**controlled Cd (II) un loaded biomass**					
**Pos. [°2Th.]**	**Height [cts]**	**d-spacing [Å]**	**Rel. Int. [%]**	**Crystallite Size only [Å]**	**Micro Strain only [%]**
**20.8837**	107.85	4.25374	62.51	920.3247	0.2311
**26.6952**	172.53	3.33944	100	927.0256	0.180116
**29.6743**	105.43	3.01062	61.11	643.4676	0.233937
**33.1376**	16.7	2.70347	9.68	410.7689	0.329074
controlled Cd (II) loaded biomass					
**Pos. [°2Th.]**	Height [cts]	d-spacing [Å]	Rel. Int. [%]	Crystallite Size only [Å]	Micro Strain only [%]
**13.8043**	40.83	4.80408	56.93	48.04762	4.99929
**20.7132**	60.13	4.21748	83.69	28.93167	7.288699
**27.7734**	89.46	3.19429	100	344.279	0.46391
**35.2557**	41.31	2.70347	9.68	410.7689	0.329074

*Where (pos.) is the position of peak (2Th), height is counts of peak (cts), and (Rel. int.) is relative intensity (%).

Table 10. EDX for controlled unloaded biomass, values of peaks showing no cadmium (II) peak for controlled cadmium (II) loaded biomass, values of peaks showing cadmium (II) peak.

unloaded biomass				
**Element**	**(KeV)**	**Mass%**	**Error%**	**Atom%**
C K	0.277	53.25	0.16	62.31
O K	0.525	39.24	0.67	34.47
Mg K	1.253	0.6	0.15	0.35
Si K	1.739	0.16	0.1	0.08
P K	2.013	3.08	0.08	1.4
S K	2.307	0.78	0.07	0.34
Cl K	2.621	0.34	0.08	0.13
K K	3.312	2.56	0.09	0.92
Total	100			
cadmium (II) loaded biomass				
**Element**	**(KeV)**	**Mass%**	**Error%**	**Atom%**
C K	0.277	10.37	0.7	19.89
O K	0.525	43.6	1.24	62.77
Mg K	1.253	0.81	0.37	0.15
Si K	1,739	0.18	0.23	0.15
P K	2.013	0.94	0.18	0.7
S K	2.307	6.31	0.14	4.53
Cl K	2.621	6.76	0.15	4.39
K K	3.312	1.18	0.2	0.7
Cd K	3.132	29.85	0.48	6.12

Table 11. Comparison between Chlamydomonas sp. as biosorbent in this study and other biosorbent in previous studies for cadmium (II).

Adsorbent	Conditions	Adsorption capacity (mg/g)	Removal efficiency %	Reference
**Orange peel (OP)-derived biochar**	Contact time: 129 min<br>Dose: 6 gm/l<br>Concentration: 100 mg/L<br>pH: 9.5<br>Temperature: 25°C	114.69	96 %	(Tran et al., 2016)
**Coated Chicken Bones with Double-Layer Hydroxide (Mg/Fe-LDH)**	Contact time: 180 min<br>Dose: 10 gm/100 ml<br>Concentration: 20 mg/l<br>pH: 5.0<br>Temperature: 25°C	-	97 %	(Alquzweeni and Alkizwini, 2020)
***Cladophora* sp.**	Contact time: 60 min.<br>Dose: 0.2 gm/l<br>Concentration: 50 mg/L<br>pH: 4.0<br>Temperature: °32 C	12.07	80 %	(Amro and Abhary, 2019)
**Natural phosphate**	Contact time: 120 min.<br>Dose: 4 gm/l<br>Concentration: 50 mg/l<br>pH: 5.0<br>Temperature: 25°C	26	-	(Yaacoubi et al., 2014)
**green synthesis iron oxide nanoparticles with tangerine peel extract**	Contact time: 90 min.<br>Dose:0. 4 gm/ 100 ml<br>Concentration: 5 mg/l<br>pH: 4.0<br>Temperature: 25°C	-	90 %	(Ehrampoush et al., 2015)
**unmodified and NTA-modified *Dendrocalamus strictus* charcoal powder**	Contact time: 120 min.<br>Concentration: 1 mg/l<br>pH: 6<br>Temperature: 25°C	166.66	91.47 %	(Saini et al., 2019)
***Chlamydomonas* sp.**	Contact time: 60 min<br>Dose: 0.8 gm/l<br>Concentration: 25 - 200 mg/L<br>pH: 4.0<br>Temperature: 25°C	44.75	95.6 %	This study
